# Diet-Induced Obesity Affects Muscle Regeneration After Murine Blunt Muscle Trauma—A Broad Spectrum Analysis

**DOI:** 10.3389/fphys.2018.00674

**Published:** 2018-06-05

**Authors:** Pengfei Xu, Jens-Uwe Werner, Sebastian Milerski, Carmen M. Hamp, Tatjana Kuzenko, Markus Jähnert, Pascal Gottmann, Luisa de Roy, Daniela Warnecke, Alireza Abaei, Annette Palmer, Markus Huber-Lang, Lutz Dürselen, Volker Rasche, Annette Schürmann, Martin Wabitsch, Uwe Knippschild

**Affiliations:** ^1^Department of General and Visceral Surgery, Ulm University Hospital, Ulm, Germany; ^2^Department of Experimental Diabetology, German Institute of Human Nutrition, Potsdam-Rehbrücke, Potsdam, Germany; ^3^Institute of Orthopaedic Research and Biomechanics, Center for Trauma Research, Ulm University Medical Center, Ulm, Germany; ^4^Core facility “Small Animal Imaging”, Ulm University, Ulm, Germany; ^5^Institute of Clinical and Experimental Trauma Immunology, Ulm University Hospital, Ulm, Germany; ^6^Division of Pediatric Endocrinology and Diabetes, Ulm University Hospital for Pediatrics and Adolescent Medicine, Ulm, Germany

**Keywords:** obesity, trauma, C57BL/6J, skeletal muscle, microarray, fibrosis, satellite cells

## Abstract

Injury to skeletal muscle affects millions of people worldwide. The underlying regenerative process however, is a very complex mechanism, time-wise highly coordinated, and subdivided in an initial inflammatory, a regenerative and a remodeling phase. Muscle regeneration can be impaired by several factors, among them diet-induced obesity (DIO). In order to evaluate if obesity negatively affects healing processes after trauma, we utilized a blunt injury approach to damage the *extensor iliotibialis anticus* muscle on the left hind limb of obese and normal weight C57BL/6J without showing any significant differences in force input between normal weight and obese mice. Magnetic resonance imaging (MRI) of the injury and regeneration process revealed edema formation and hemorrhage exudate in muscle tissue of normal weight and obese mice. In addition, morphological analysis of physiological changes revealed tissue necrosis, immune cell infiltration, extracellular matrix (ECM) remodeling, and fibrosis formation in the damaged muscle tissue. Regeneration was delayed in muscles of obese mice, with a higher incidence of fibrosis formation due to hampered expression levels of genes involved in ECM organization. Furthermore, a detailed molecular fingerprint in different stages of muscle regeneration underlined a delay or even lack of a regenerative response to injury in obese mice. A time-lapse heatmap determined 81 differentially expressed genes (DEG) with at least three hits in our model at all-time points, suggesting key candidates with a high impact on muscle regeneration. Pathway analysis of the DEG revealed five pathways with a high confidence level: myeloid leukocyte migration, regulation of tumor necrosis factor production, CD4-positive, alpha-beta T cell differentiation, ECM organization, and toll-like receptor (TLR) signaling. Moreover, changes in complement-, Wnt-, and satellite cell-related genes were found to be impaired in obese animals after trauma. Furthermore, histological satellite cell evaluation showed lower satellite cell numbers in the obese model upon injury. *Ankrd1, C3ar1, Ccl8, Mpeg1*, and *Myog* expression levels were also verified by qPCR. In summary, increased fibrosis formation, the reduction of *Pax7*^+^ satellite cells as well as specific changes in gene expression and signaling pathways could explain the delay of tissue regeneration in obese mice post trauma.

## Introduction

The skeletal muscle exhibits a high potential to regenerate after injury, although the total turnover rate of the muscle is very low. Muscle regeneration can be divided into the initial response phase, which includes early inflammation and degenerative events, the peak inflammation, and degenerative response phase, and the structural and functional muscle recovery phase. The complement system as major fluid phase of the innate immune system is crucially involved in the initiation of the inflammatory phase. It is rapidly activated in injured muscle tissue leading to immune cell infiltration in the lesion site (Frenette et al., 2000). Neutrophils are the first to be recruited. Thereafter, monocytes invade the lesion area and differentiate into pro-inflammatory M1 macrophages (M1Φ). Resident MΦ are activated to remove tissue debris, attract satellite cells to the injured site, and stimulate their proliferation (Tidball and Villalta, 2010; Rigamonti et al., 2014). In later stages, M2 macrophages (M2Φ; 24–48 h) resolve inflammation. In response to injury, satellite cells (*Pax7*^+^ progenitor cells), which can be found in a niche between sarcolemma and basal lamina, are activated (as documented by the expression of the myogenic regulatory factors *Myf5* and *Myod*), transiently proliferate and upregulate genes necessary for terminal differentiation (myogenin and *MRF4*) (Chargé and Rudnicki, 2004). There is only minor evidence of a direct contribution to the production of new muscle fibers by other cells residing in the interstitium or recruited from a distal source. Thus, research has focused on understanding the processes within the microenvironment that modulate satellite cell renewal and differentiation, including the composition and milieu secreted by both inflammatory and non-inflammatory cells (Boppart et al., 2013; Yin et al., 2013).

MΦ contribute to muscle regeneration by stimulating survival and proliferation of primary progenitor cells in the muscle, the satellite cells (*Pax7*^+^) (1–8 days) (Cantini et al., 1994; Warren et al., 2005), by releasing pro-inflammatory cytokines (Li, 2003; Toth et al., 2011) and by promoting myogenic differentiation through M2Φ. In addition, fibroblasts are also involved in regulating satellite cell proliferation and differentiation (Murphy et al., 2011). However, the complex fine-tuned regeneration process of injured muscles can be perturbed by several factors, such as obesity (Finkelstein et al., 2007; Norton et al., 2011; Abhyankar et al., 2012; Nelson et al., 2012). In obesity, adipose tissue functions are deregulated leading to changes in the release of growth factors, adipocytokines, cytokines, chemokines, hormones, and fatty acids which are secreted from adipocytes and MΦ resident in white adipose tissue (Fischer-Posovszky et al., 2007; Wozniak et al., 2009; Adamczak and Wiecek, 2013; Al-Suhaimi and Shehzad, 2013; de Oliveira Leal and Mafra, 2013; Cao, 2014). These changes affect lipid metabolism, glucose homeostasis, inflammation, angiogenesis, hemostasis, and blood pressure. In addition, the ectopic lipid accumulation in several organs and tissues can lead to various severe co-morbidities including heart disease, diabetes, metabolic syndrome, hypertension, sleep apnea, and cancer.

However, little is known on the molecular level whether and how early lifetime nutrition and severe obesity influence the regeneration of injured muscles (Trayhurn et al., 2011; Woo et al., 2011; Akhmedov and Berdeaux, 2013). The role of obesity on skeletal muscle physiology, structural maintenance, and regeneration after exposure to myotoxins or freezing has been analyzed in several obese animal models, such as: leptin-deficient (ob/ob), leptin receptor-deficient (db/db) mice, POUND (Lepr^db/lb^) mice lacking all functional leptin receptors, obese Zucker rats bearing a mutation in the leptin receptor, as well as in rodents fed a high-fat diet (HFD). In normal animals, myotoxin- or freezing-induced injury causes local myofiber necrosis and inflammation, followed by satellite cell activation, proliferation, differentiation, fusion, and ultimately regrowth of myofibers to approximate the same size as the original within 3 weeks (Chargé and Rudnicki, 2004). In contrast, regeneration of injured skeletal muscle is impaired in obese mice: Compared to normal weight littermates HFD mice showed a reduction in muscle mass associated with smaller myofibrils, larger interstitial spaces, increased collagen deposition, and a reduction in the amount and regeneration ability of satellite cells (Hu et al., 2010; Woo et al., 2011). These findings suggest that elevated levels of lipid metabolites, pro-inflammatory cytokines as well as insulin resistance impair satellite cell function in obese mice, finally leading to decreased regeneration capacity (Bosma et al., 2012; Coen and Goodpaster, 2012; Akhmedov and Berdeaux, 2013). However, the exact mechanisms how obesity impairs cellular processes during the different stages of repair, have to be elucidated.

Of note, a model combining HFD with an injury is rare although of high clinical relevance. The most common types of muscle trauma models apply freezing, barium chloride, notexin, or cardiotoxin (Hardy et al., 2016). Depending on the injury type, a different response with regard to regeneration will take place. Since the physical approach for a blunt muscle injury is grossly underrepresented in current literature, we aimed at using a drop tower model for application of injury.

Here, we continued to further analyze the role of obesity on muscle regeneration using our drop-tower based model (Werner et al., 2018) for induction of a blunt trauma to the *extensor iliotibialis anticus* muscle of normal weight and obese mice. Imaging the trauma induction and regeneration process by magnetic resonance imaging (MRI) clearly showed edema formation and hemorrhage exudate in muscles of normal weight and obese mice. Morphological analysis revealed the occurrence of tissue necrosis, immune cell infiltration, extracellular matrix (ECM) remodeling and fibrosis formation in the damaged muscle. Regeneration was delayed in muscles of obese mice, with a higher incidence of fibrosis formation due to hampered expression levels of underlying genes. To further analyze previously obtained microarray data (Werner et al., 2018) a time lapse heatmap for an unbiased evaluation of striking candidates was generated. With this approach, we identified 81 differentially expressed genes (DEG) associated, but not limited to, five pathways, which play crucial roles during regeneration. In addition, we focused on Wnt- and satellite cell-related genes in order to assess the influence of obesity on muscle regeneration after injury. Whereas, histological analysis confirmed the notion, that satellite cell numbers are impaired upon injury in obese mice, the detected differences in expression levels of *Ankrd1, C3ar1, Ccl8, Mpeg1*, and *Myog* in normal and obese mice could be verified by qPCR. Summarizing, obese mice showed a general lack of regenerative response at all-time points compared to their normal weight counterparts, thus further illustrating the negative influence of obesity on the healing process after skeletal muscle injury.

## Materials and methods

### Animal housing, breeding, sampling, and treatment

C57BL/6J mice were purchased at the age of 16 weeks from the in-house breeding facility within the Animal Research Center at Ulm University and kept in a pathogen free open cage facility pre-injury and in an IVC cage facility post-injury. Animals were kept in a 12 h light/dark cycle at 22.5 ± 1°C with access to food and water *ad libitum*.

C57BL/6J mice were used for breeding purposes, whereas parent animals received either normal diet (ND; 10% kcal fat; D12450J) or HFD (60% kcal fat; D12492) 1 week prior in order to influence the prenatal development of litters until breeding possibility was exhausted by regularity. Both diets were purchased from Research Diets Inc., by their European distributor Brogaarden® in Gentofte, Denmark. Litters were weaned after 3 weeks and received their respective parental diet for 16 ± 1 weeks until they were sacrificed. Female litter were used for the presented experiments. All animal experiments were approved by the local and state authorities (license number: 1183) and carried out in accordance with local regulations and ARRIVE guidelines. In order to assess the progression of injury in normal weight and obese C57BL/6J mice by immunohistochemistry analysis, gene expression profiling and RT-qPCR, analyses were performed to assess muscle regeneration at 1, 6, 24 h, 3 days, and 8 days post injury. In addition, MRI, HE-, and Sirius Red stainings were also performed 21 days after trauma induction. For this purpose, female C57BL/6J litter animals were randomly grouped into sham and treated groups. Induction of injury to *extensor iliotibialis anticus* muscle of the left hind leg was carried out by using a physical trauma model previously described (Claes et al., 2006; Weckbach et al., 2013; Wanner et al., 2017; Werner et al., 2018). Briefly, anesthesia was carried out with 2.5 vol% sevoflurane (Sevorane™ Abbott, Wiesbaden, Germany) mixed with 97.5 vol% oxygen. Analgesic treatment involved s. c. injection of Buprenorphine [Temgesic® Reckitt Benckiser, Berkshire, Great-Britain (0.03 mg/kg KG)] 30 min before injury. A weight (40 g) was dropped from a defined height (120 cm bar length; 100 cm effective case depth) on the muscle, generating a defined force input to damage the muscle. Bone fracture was avoided by using a spacer (3 mm), in order to limit the depth of penetration (see Supplementary Figure 1). For increased reproducibility, the area around the muscle to be hit was shaved and marked. Control animals received the same procedure without application of injury. At the defined time points, mice were sacrificed by CO_2_ inhalation following standard procedure. Tissue was isolated, snap frozen in liquid nitrogen, and stored at −80°C until further use. Additionally, tissue was also formalin-fixed and embedded in paraffin.

### Determination of force input into *extensor iliotibialis anticus*

In order to evaluate whether both, normal weight and obese mice were subjected to a similar force input, the impact force was assessed and calculated by measuring the travel of the trauma wedge over time by using a laser distance sensor (OptoNCDT 2200, Micro-Epsilon, Germany). A more detailed description can be found in Wanner et al. (2017). For this, nine female C57BL/6J mice receiving either a normal or HFD were used.

### *In vivo* imaging

Muscle damage was assessed by *in vivo* MRI. All data were acquired on a dedicated ultrahigh field 11.7T small animal system (BioSpec 117/16, Bruker Biospin, Ettlingen, Germany) equipped with a 9 cm gradient insert (BGA-S9) operating with ParaVision 6.01 software. Signal was received with a four-channel receive-only surface coil placed anterior to the TA. During scanning the respiratory frequency and body temperature were recorded with a MR compatible small animal unit (SA Instruments, Inc., NY, USA). For reproducible positioning, the mouse was placed in supine position with both legs parallel to the bore of the magnet in a self-made holder. Anesthesia was maintained using 1.5% isoflurane (abbvie, Wiesbaden, Germany) and was adjusted to maintain a safe respiration rate about 80 cycles per minute. All animals were scanned prior to the injury and 1, 6, 24 h, 3 days, 8 days, and 21 days after injury.

The following MR scans were performed: 2D FLASH in coronal and axial slice orientation with acquisition parameters as TE/TR = 3.1/280 ms, spatial resolution Δ*r* = 100 × 100 × 1,000 μm3; T1-weighted multi spin-echo (T1-RARE) with acquisition parameters as TE/TR = 12.4 /815 ms, Δ*r* = 86 × 100 × 500 μm3, RARE factor = 3; T2-weighted multi spin-echo (T2-RARE) with acquisition parameters as TE/TR = 46.5/3,470 ms, Δ*r* = 86 × 100 × 500 μm3, RARE factor = 16, and a multi spin-echo T2 mapping sequence acquiring 30 echoes with 7.5 ms echo spacing with Δ*r* = 171 × 200 × 500 μm3. T2 mapping was performed using a mono-exponential fit with offset correction.

### Hematoxylin and eosin (HE) staining

Muscle tissue from female normal weight (*n* = 3) and obese mice (*n* = 3) was collected before and 1, 6, 24 h, 3, 8, and 21 days after trauma induction and subsequently embedded in paraffin. 5 μm thick tissue-sections were deparaffinized, rehydrated, and stained by HE as described previously (Werner et al., 2018).

### Sirius red staining

Paraffin embedded *Extensor iliotibialis anticus* muscle tissue from normal weight (*n* = 3) and obese mice (*n* = 3) collected before and 1, 6, 24 h, 3, 8, and 21 days after trauma induction was longitudinally cut into 5 μm-thick sections with circular layer and myenteric ganglia for cross-sections. Deparaffinized sections were stained with 0.1% Sirius Red dissolved in aqueous saturated picric acid for 1 h. Thereafter, sections were washed twice in water, and subsequently stained with hematoxylin for 10 min. Dehydration was carried out by incubation of the sections in 96% EtOH twice for 5 min, followed by incubation in 100% EtOH twice for 5 min and twice in Roti®-Histol for 10 min. Finally, sections were mounted using Entellan®.

### Immunohistochemical analysis of Pax7, C3ar, and C5ar

Deparaffinized and rehydrated 3 μm thick muscle sections collected before at the indicated time points after induction of normal weight and obese mice were microwaved with CitraPlus solution (Biogenex, San Ramon, California, USA) for antigen retrieval. Sections were then incubated in 3% H_2_O_2_ for 15 min to prevent endogenous peroxidase activity, whereas unspecific binding sites were subsequently blocked by applying blocking solution (TBS + 0.3% Tween20 + 10% goat serum) at RT for 30 min. Anti-Pax7 (ThermoFisher Scientific, Dreieich, Germany; PA1-24471; 1:750; *n* = 6), anti-C3ar (Bioss Antibodies; Woburn, MA, USA; bs-2955R; 1:100; *n* = 3) or anti-C5ar (proteintech®; Manchester, United Kingdom; 21316-1-AP; 1:100; *n* = 3) were applied and incubated at RT for 2 h. Goat anti-rabbit secondary antibodies (N-histofineH from Nichirei Corporation, Tokyo, Japan for Pax7; Jackson ImmunoResearch Laboratories Inc., West Grove, PA, USA; 111-035-144; 1:50; for C3ar/C5ar) were then added for 1 h at RT and developed by adding the chromogen 3,39-diaminobenzidine (DAB; Agilent, Santa Clara, CA, USA). Sections were counterstained with hematoxylin and briefly immersed into 1% HCl in EtOH to prevent unspecific binding. Sections were then de-hydrated and mounted using Entellan® (Merck, Darmstadt, Germany).

### RNA isolation and microarray analysis

**Figure 1:**
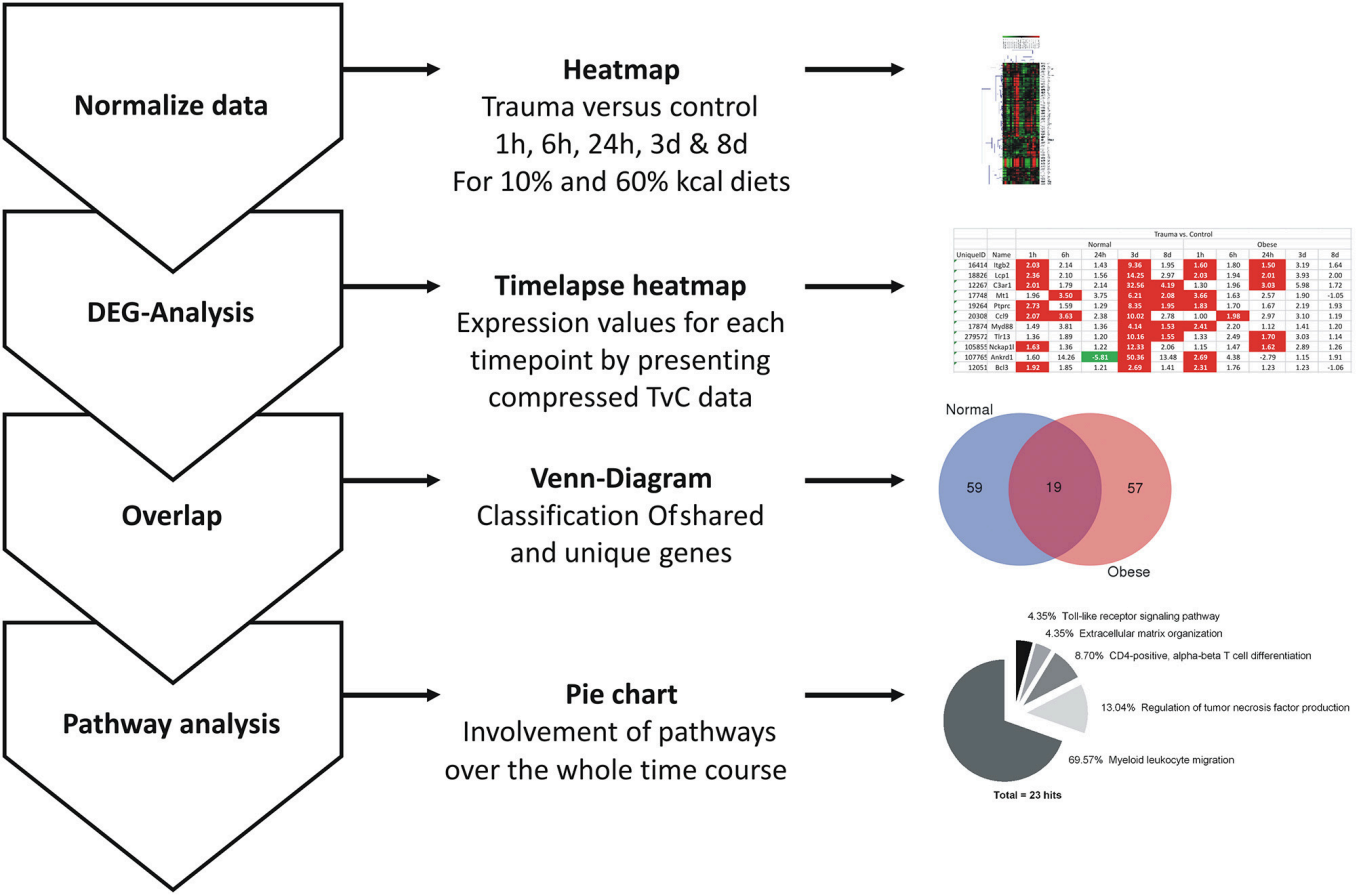
General workflow for evaluation of present microarray data. After normalization of data and generation of heatmaps for each comparison, a general DEG analysis per time point and diet for the generation of a time-lapse heatmap was carried out. The total amount of genes for normal weight and obese mice at each time was compared to screen for unique and shared genes using Venn diagrams at the indicated time points. Final pathway analysis was carried out with ClueGO (Bindea et al., 2009), a plugin for Cytoscape (Lotia et al., 2013).

Tissue from *extensor iliotibialis anticus* muscle of normal weight and obese female mice, control and injury (*n* = 3 per group), was used. Harvesting time points were 1, 6, 24 h, 3, and 8 days to cover the early and middle stage regeneration process. Isolation of total RNA, microarray analysis and evaluation method were described previously (Werner et al., 2018). Briefly, CEL-files of trauma vs. control were compared per time point and diet, respectively. While each time point could be used to generate a traditional heatmap, we comprised the data previously published by Werner et al. into a time lapse (Werner et al., 2018), screened shared and unique genes, and performed a pathway analysis with the high impact genes (81 in total). The general workflow of this method is shown in Figure 1. Full data can be found at Gene Expression Omnibus under the acronym “GSE103726.”

### Real-time qPCR

Hundred nanograms of total RNA was transcribed into cDNA using the AffinityScript cDNA Synthesis Kit (Agilent Technologies, Santa Clara, USA) according to manufacturer's instructions. *Ppia* (*Mm_Ppia_1_SG QT00247709*) was used as reference gene following the recommendations of Gong et al. (2016). Marker genes were *Ankrd1* (*Mm_Ankrd1_1_SG QT00104419), C3ar1* (*Mm_C3ar1_1_SG QT00251216*), *Ccl8* (*Mm_Ccl8_1_SG QT00128548*), *Mpeg1* (*Mm_Mpeg1_1_SG QT00247660*), and *Myog* (*Mm_Myog_1_SG QT00112378*). Reference genes, markers and SYBR® Green (QantiTect® SYBR® Green PCR Kit) were purchased from Qiagen (Hilden, Germany). Time points evaluated were 24 h, 3, and 8 days for sham and trauma animals (*n* = 3). Data evaluation was carried out as follows: Each well was related to the average of the housekeeping gene, then averaged again for one value per mouse. Mice were grouped appropriate to time of treatment (control/injury) and diet. Each injured subgroup was normalized on their corresponding controls to determine x-fold values.

### Statistical evaluation

Statistical analysis was carried out by two-sided homoscedastic *t*-test, for weight distribution, force input, satellite cell counts, and qPCR. Calculation of microarray data was performed by R with the help of the “questionr” package (see Supplementary Table 1). Microarray evaluation used the *t*-test function of R to determine significance. Briefly, the *t*-test function performs a two-sided *t*-test. However, as the *t*-test function arguments by default are “paired = FALSE, var.equal = FALSE” it instead switches to a Welch-test (or unequal variances *t*-test), which neither needs equal sample sizes nor assumes equal variances, while solely focusing on the differences between the averages μ1 and μ2 for each group.

### Software

Figures were generated using GraphPad Prism version 5 for Windows, GraphPad Software, San Diego California USA, www.graphpad.com.

Analysis of CEL files was carried out with RStudio (RStudio Team, 2016) (version 0.99.903), R (R Development Core Team, 2016) (version 3.2.5), and several R-packages (e.g., “affy,” “pvclust,” “gplots,” and “FactoMineR”). The complete list of packages, including version number, can be found in Supplementary Table 1.

Pathway analysis was performed using ClueGO (Bindea et al., 2009) v2.3.3, a plug-in for Cytoscape (Lotia et al., 2013).

## Results

In order to assess the influence of obesity on blunt muscle injuries, an established drop tower device was used for trauma induction on the muscle *extensor iliotibialis anticus* on the left hind leg of 16-weeks old C57BL/6J mice fed either with a 10 or 60% kcal diet (Claes et al., 2006; Weckbach et al., 2013; Werner et al., 2018). Characteristics of animals and experimental details of trauma induction are depicted in Table 1.

**Table 1:** Characteristics of C57BL/6J mice and for the induction of a blunt muscle injury.

**ANIMALS**				
Strain	C57BL/6J			
Sex	Female			
Diet	D12450J		D12492	
	10% kcal fat		60% kcal fat	
Time points	1, 6, 24 h, 3, and 8 days			
Replicates	3 per time point			
Total amount	60			
**BLUNT MUSCLE INJURY**				
Anesthesia	Sevorane™ [2.5 Vol%]			
Analgesic	Temgesic^®^ [0.03 mg/kg KG]			
Weight	40 g			
Guiding rod (total length)	120 cm			
Guiding rod (effective drop height)	104 cm			
Spacer	3 mm			
Target	*Extensor iliotibialis anticus*			
Group	Normal		Obese	
Treatment	Control	Trauma	Control	Trauma
Age (weeks)	16.1 ± 0.52	16.2 ± 0.56	16.2 ± 0.80	16.3 ± 0.92
Body weight (g)	20.6 ± 1.30	21.8 ± 1.49	29.7 ± 4.48	29.7 ± 4.36

*Shown are diet, strain, sex, treatment, sample size, age (±sd), and body weight (±sd)*.

### Morphological evaluation of a blunt muscle injury to *extensor iliotibialis anticus*

**Figure 2:**
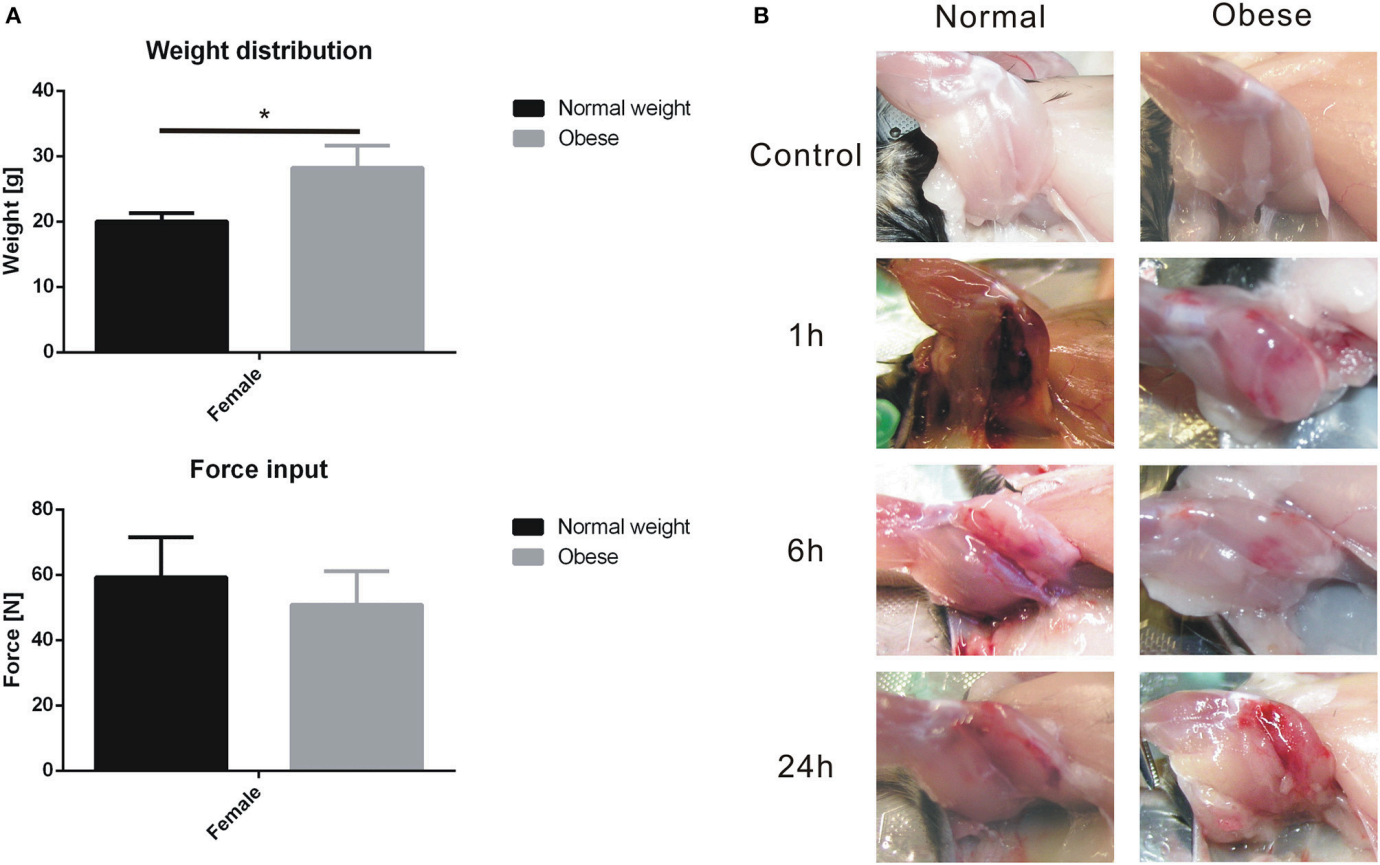
Body weight, force input, and soft tissue trauma induction of the left hind leg of C57BL/6J mice. Values are given with mean ± standard deviation; *n* = 9. Statistical analysis by two-sided homoscedastic *t*-test. **(A)** Weight distribution for normal weight and obese animals (^*^indicates *p* ≤ 0.05); Force input into e*xtensor iliotibialis anticus*, values showed no significance. **(B)** Left hind limb of normal weight and obese C57BL/6J mice before and after trauma induction. Hematoma formation was observed within 24 h.

Although weight distribution of normal weight and obese mice showed significant differences within the control and treated group (Figure 2A), no statistical significant weight-specific differences in the impact forces (Figure 2A) created by the drop tower device were observed. Here, the average forces ranged between 40 and 75 N for our animals, regardless of diet. Hematoma formation and blood inclusion in injured muscle tissue was visible in each case, whereas hematoma formation increased within the first 24 h (Figure 2B).

**Figure 3:**
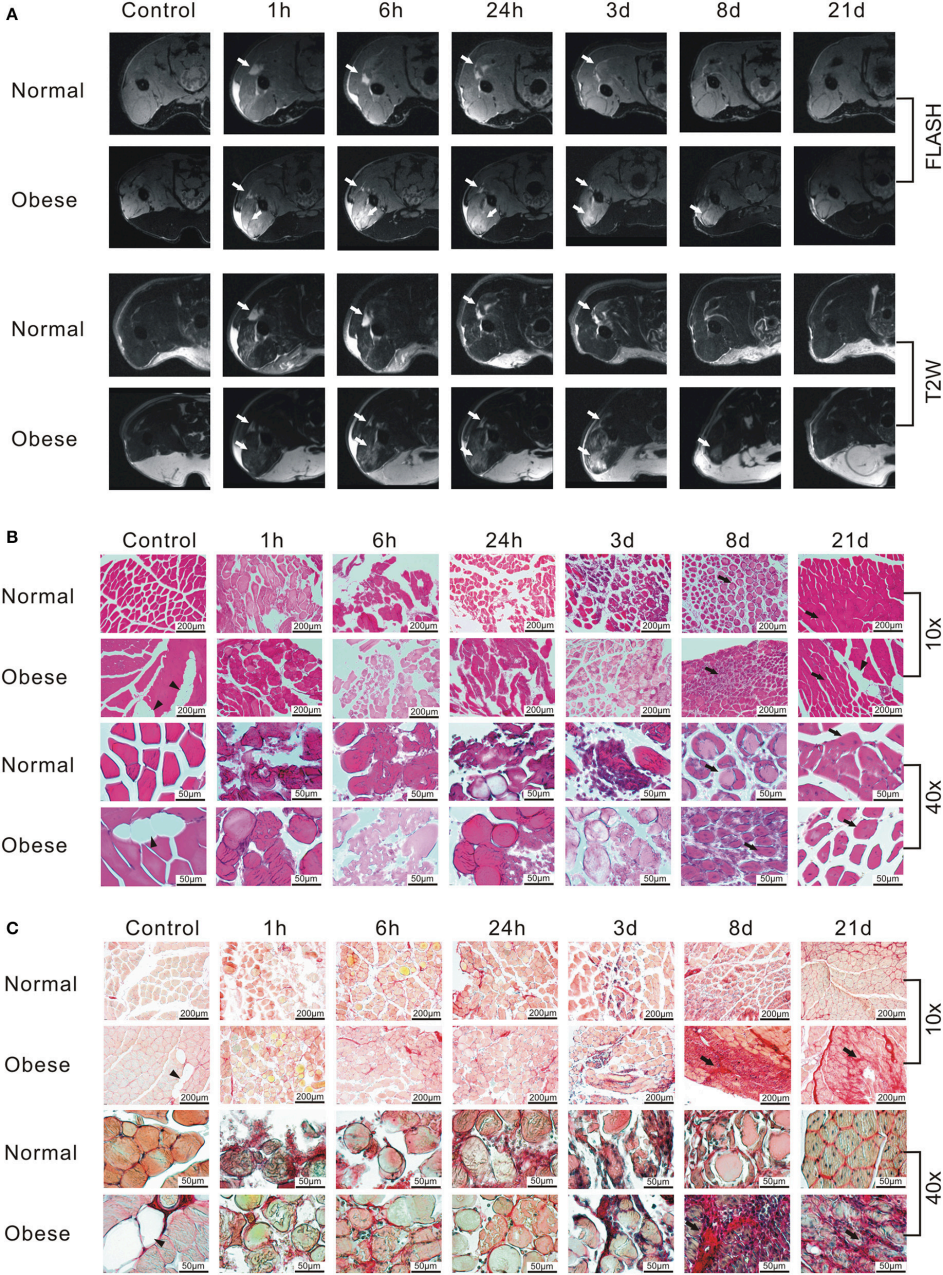
Monitoring of the regeneration process after induction of a blunt muscle injury in female normal weight and obese C57BL/6J mice. **(A)** FLASH and T2W-RARE recordings of two female C57BL/6J mice receiving either ND or HFD. Arrow = edema. Same animals were used for the whole time course. Prescan (Control) did not show any signs of injury, whereas an edema can be seen starting 1 h post-injury. Edema formation increases until 6 h post-injury, then recedes within 21 days, whereas the obese mouse shows signs of edema 8 days after injury. The normal weight mouse seems to recover within the observed time frame. **(B)** Hematoxylin-eosin (HE) staining of muscle tissue sections. The staining shows damaged muscle, inflammatory cells and subsequent myofiber regeneration within 21 days post-injury. Triangle = fat inclusion, arrow = newly regenerated myofiber. **(C)** Sirius Red staining of muscle tissue sections. Triangle = fat inclusion, arrow = fibrosis. Pictures were taken with Olympus IX81 using Xcellence v.1.2. Scale = 200 μm at 10x magnification; Scale = 50 μm at 40x magnification.

Analysis of physiological changes in the injured leg of female normal weight and obese mice by MRI at different time points of the regeneration process revealed the occurrence of interstitial fluid, edema, and hemorrhage exudate along the gap between muscle fibers and muscle bundles in both, FLASH and T2W images (Figure 3A). Edema formation increased until 6 h post-injury, then receded within 21 days. However, the obese mouse still showed signs of edema 8 days after injury, while the normal weight mouse seemed to recover. Next, to assess general morphological changes in paraffin-embedded muscle tissue of female, normal weight and obese mice at different time points of the regeneration process, HE staining of muscle tissue was conducted at the indicated time points (Figure 3B). Morphology of muscle tissue of control animals was similar, except for the increased fat incorporation in the tissue of obese mice. One hour post-injury, the disruption of muscle tissue and the presence of erythrocytes in normal weight and obese mice were visible. Six hour post-injury immune cells started to infiltrate the damaged tissue peaking at 24 h in normal weight mice, and at day 3 in obese mice. Tissue debris was removed in the muscle tissue of normal weight mice 8 days post-injury, while muscle tissue of obese just showed first signs of beginning regeneration. Twenty-one days post-injury muscle tissue of normal weight animals was almost fully regenerated, whereas the regeneration process in muscle tissue of obese mice was still incomplete and accompanied with a higher rate of fat incorporation. To detect changes in the amount of connective tissue, Sirius Red staining was performed (Figure 3C). In normal weight and obese sham animals, healthy muscle fibers with small amounts of connective tissue were observed in cross sections. However, cross sections of obese animals showed a higher incorporation of fat than those of normal weight animals. Whereas, no distinct differences in the degree of fibrosis were detectable until day 3 post injury, distinct changes in the degree of fibrosis were observed 8 days post-injury. Overall, obese animals showed more severe cases of fibrosis when compared to their normal weight counterparts. These changes manifested in obese mice 21 days post injury.

Following our histochemical findings in fibrous tissue, we focused on identifying genes involved in fibrosis formation during regeneration of injured muscles. Here, normalized relative gene expression levels (“Trauma vs. Control”) were compared between normal weight and obese mice at 1, 6, 24 h, 3, and 8 days after trauma induction (Table 2). Alterations were detected in collagen-, fibronectin-, TGF-, IL-, MMP-, TIMP-, and Plasminogen-related families. Whereas, expression levels of collagen type I, III, V, VI, VIII, XII, XIV, and fibronectin increased up to 3 days post injury in normal weight mice, obese animals seem to lack a response to injury. Furthermore, expression levels of additional factors involved in ECM remodeling, among them *Plat, Plau, Il10ra, Il10rb, Il13ra1, Tgfb1, Tgfbr2, Mmp2, Mmp3, Mmp14, Timp1*, and *Timp2* were increased in normal mice after injury, but much weaker effects were observed in obese mice.

**Table 2:** Gene expression levels in fold change values determined by microarray analysis for fibrosis formation.

**ID**	**Name**	**Trauma vs. Control**									
		**Normal**					**Obese**				
		**1 h**	**6 h**	**24 h**	**3 days**	**8 days**	**1 h**	**6 h**	**24 h**	**3 days**	**8 days**
**PRO-FIBROTIC**											
11475	*Acta2*	1.25	−1.14	1.08	**3.14**	1.16	−1.05	1.13	1.06	−1.15	1.15
11606	*Agt*	1.06	1.15	1.27	−**1.92**	−1.33	1.58	1.10	−**1.11**	−1.08	−1.23
20302	*Ccl3*	2.46	7.16	1.24	5.45	**1.18**	**3.93**	7.50	**1.40**	2.00	1.13
14219	*Ctgf*	**2.35**	2.35	−**1.96**	**3.70**	1.36	1.91	1.92	−1.15	−1.15	−1.09
**ANTI-FIBROTIC**											
16154	*Il10ra*	−1.15	1.12	1.07	**3.21**	1.46	**1.19**	1.02	**1.16**	1.39	1.06
16155	*Il10rb*	1.28	1.03	−1.16	**5.45**	1.75	1.10	1.15	1.11	1.89	1.25
16164	*Il13ra1*	1.37	1.89	1.06	**4.96**	1.66	1.22	2.56	1.12	1.10	1.25
**COLLAGEN COMPONENTS**											
12816	*Col12a1*	1.69	1.43	−**1.97**	4.67	**2.47**	1.27	1.17	−1.37	1.25	1.08
12818	*Col14a1*	1.44	1.14	−1.02	2.30	**2.88**	−1.34	−1.03	1.18	1.31	1.53
12842	*Col1a1*	1.54	1.35	−1.02	**5.74**	**4.43**	−1.03	−1.07	1.20	2.96	1.23
12843	*Col1a2*	1.49	1.29	−**1.20**	**4.55**	**3.24**	−1.06	−1.04	1.18	2.32	1.13
12825	*Col3a1*	**1.53**	1.14	**1.66**	**4.59**	**3.71**	−1.26	1.04	**1.38**	2.65	1.48
12832	*Col5a2*	1.26	1.15	1.11	**3.36**	**2.50**	1.05	1.06	**1.12**	1.88	1.18
12833	*Col6a1*	1.30	1.08	**1.11**	**2.22**	1.86	−1.11	−1.03	**1.24**	1.53	1.14
12835	*Col6a3*	1.17	1.25	**1.40**	**2.31**	**2.18**	−1.26	1.08	**1.42**	1.71	1.21
12837	*Col8a1*	1.14	−1.03	−1.17	4.60	**1.99**	1.06	1.14	−1.21	1.23	−1.01
*14268*	*Fn1*	1.82	1.35	1.00	**6.07**	**3.39**	−1.11	1.16	1.04	1.18	**1.51**
**REMODELING ENZYMES**											
16948	*Lox*	**2.53**	3.12	−1.14	**12.02**	**2.36**	1.34	2.17	2.06	3.64	1.22
268977	*Ltbp1*	1.14	1.18	−1.06	**2.27**	1.62	1.01	−1.01	1.06	1.04	1.10
17387	*Mmp14*	1.32	1.37	1.03	**3.40**	2.80	1.11	1.34	1.00	1.77	1.31
17390	*Mmp2*	1.19	1.03	−1.23	**1.77**	**2.63**	−1.07	−1.17	−1.00	1.25	1.23
17392	*Mmp3*	1.16	2.97	1.92	1.15	1.28	−1.14	1.94	**2.19**	2.28	1.14
18791	*Plat*	1.23	1.19	−1.32	**2.59**	1.47	1.17	1.29	1.01	1.06	1.29
18792	*Plau*	1.13	1.15	−1.00	2.77	1.16	1.09	**1.32**	1.06	1.50	1.02
18787	*Serpine1*	**4.44**	3.18	1.18	**9.62**	1.19	**3.69**	3.30	1.19	1.74	1.06
20720	*Serpine2*	−1.14	1.01	−1.03	−**1.94**	−**1.75**	1.05	−1.05	−**1.13**	−1.15	−1.07
21857	*Timp1*	1.14	4.14	1.43	**19.05**	1.91	1.74	**3.79**	1.52	2.66	1.09
21858	*Timp2*	1.17	1.05	−**1.34**	**2.04**	**1.61**	−1.06	−1.09	1.03	1.09	1.05
**OTHER REGULATORY FACTORS AND MARKERS**											
12111	*Bgn*	1.16	−1.01	−1.14	**4.81**	**2.31**	1.05	−1.03	−1.06	2.07	1.07
12153	*Bmp1*	1.17	1.06	1.01	**2.23**	1.71	1.05	−1.00	−1.01	1.25	1.15
18034	*Nfkb2*	1.15	1.34	1.08	**2.10**	1.80	1.20	1.17	1.03	1.13	1.05
18542	*Pcolce*	−1.06	1.11	1.26	**2.56**	1.60	−1.20	−1.11	1.45	1.37	−1.03
21425	*Tfeb*	−1.14	1.01	**1.24**	−**1.53**	−**1.37**	−1.00	−1.13	1.04	1.28	−1.05
21803	*Tgfb1*	1.19	1.52	1.30	**5.10**	3.23	1.18	1.40	**1.26**	1.86	1.11
21813	*Tgfbr2*	1.22	1.29	−1.08	**2.25**	**1.66**	1.01	1.23	1.05	1.22	1.02
21838	*Thy1*	1.10	1.25	−1.01	**3.98**	2.11	1.02	−1.05	1.01	1.40	1.29
21897	*Tlr1*	1.02	1.15	1.28	**2.58**	**1.24**	−1.07	1.13	1.11	1.54	1.11
279572	*Tlr13*	**1.36**	1.89	1.20	**10.16**	**1.55**	1.33	2.49	**1.70**	3.03	1.14
24088	*Tlr2*	1.74	1.12	1.06	**2.05**	**1.32**	1.49	1.33	1.05	1.22	1.06
21898	*Tlr4*	1.74	1.61	−1.40	**3.70**	2.20	1.16	1.32	−1.03	1.48	1.12
170743	*Tlr7*	1.31	1.22	1.12	**10.26**	**1.82**	1.06	1.07	1.54	2.60	1.07
170744	*Tlr8*	1.27	**1.61**	1.28	**5.90**	1.54	−1.22	1.26	1.16	2.60	1.18
81897	*Tlr9*	1.15	1.15	1.12	**3.32**	1.31	−1.11	1.12	**1.26**	1.63	1.05

*Red = significantly upregulated, green = significantly downregulated. Significance level is p ≤ 0.05. Log2 values were converted to fold change, and subsequently if value < 1 inverted to get negative values, thus baseline = ±1. Values with at least one significant value ≥ ± 1.5 are shown. For corresponding significance levels, see Supplementary Table 2*.

### Gene cluster differences of muscle tissue in normal weight vs. obese mice after muscle trauma

While we first specifically focused on genes involved in fibrosis formation, an unbiased approach to determine genes with highest impact on muscle regeneration up to 8 days was applied. For this purpose, expression data of each time point (1 h to 8 days) per diet was normalized by Robust Multi-array Average for trauma vs. control animals, respectively, consequently allowing the compression of our data for time and diet, finally resulting in only one table (viewed as a heatmap) instead of 10 traditional heatmaps. Our results clearly revealed the influence of injury on our respective diet model. In order to assess DEG, *p*-values were determined at *p* ≤ 0.05. Hierarchical cluster analysis (HCA), principal component analysis (PCA), and volcano plots were calculated by their respective functions within our script for microarray evaluation.

HCA showed a clear distinction in the cluster dendrogram, in which the combination of data sets connected in a cluster can be exchanged with the next level for each time point. Sham and trauma groups within differently expressed genes for normal weight and obese mice can be clearly distinguished at all-time points in both, approximately unbiased, marked red, and bootstrap probability, marked green (Supplementary Figure 2). PCA also resulted in the separation of control and trauma mice in DEG over the treatment period for normal weight and obese mice, indicating that both groups are not related. The first principal component usually covered around 80% of all variables, while the second principal component had less influence of max. 5–6% (Supplementary Figure 3). Volcano plots were generated albeit considering a significance level of *p* ≤ 0.05 and a log fold change (lfc) of at least ±0.75 in a log2 scale. Overall data indicated that normal weight animals show higher numbers of genes being up- and downregulated at the analyzed time points. Examples were *Serpine1* (1 h), *Serpinb1a* (24 h, 8 days), *Ankrd1* (24 h), *Ccr2* (3 days), *Serp1* (3 days), and *Slc37a4* (3 days) in muscle tissue of normal weight animals and *Fosb* (1 h), *Egr1* (1 h), *Lrrc32* (6 h), *Ms4a4c* (24 h), *Mpeg1* (24 h), *Fmod* (24 h), *Ostn* (3 days), *Myl* (3 days), and *Socs2* (8 days) in muscle tissue of obese animals. Interestingly, obese animals showed either a delay, or a complete lack of response, whereas normal weight mice showed a relative strong counter mechanism at 24 h and 3 days post-trauma (Supplementary Figure 4).

### Reduction of the quantitative gene response to muscle trauma in obese mice

**Figure 4:**
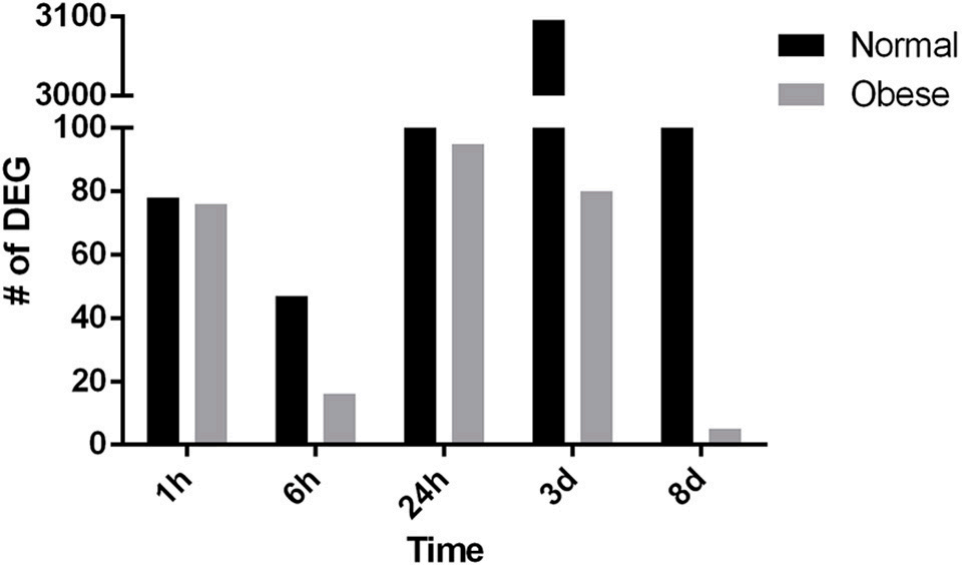
Differentially expressed genes in normal weight and obese mice respectively (*p* ≤ 0.05; fold change ≥ ± 1.5).

Next, for comparative reasons, the total amount of DEG was counted for both, normal weight and obese mice at the indicated time points (Figure 4). Strikingly, with regard to the total amount of DEG, normal weight animals showed a defined response to injury peaking 3 days post-injury. However, obese animals lacked a distinct response to injury in our model, which was especially obvious 3 days post-injury where 3,096 genes in normal weight compared to 80 genes in obese mice were found. Overall, obese animals showed lower numbers of DEG at all-time points, thus clearly indicating a lack of response to trauma despite showing a distinct response in the tissue.

### Minimal overlap of regulated genes after muscle trauma in obese vs. normal weight mice

**Figure 5:**
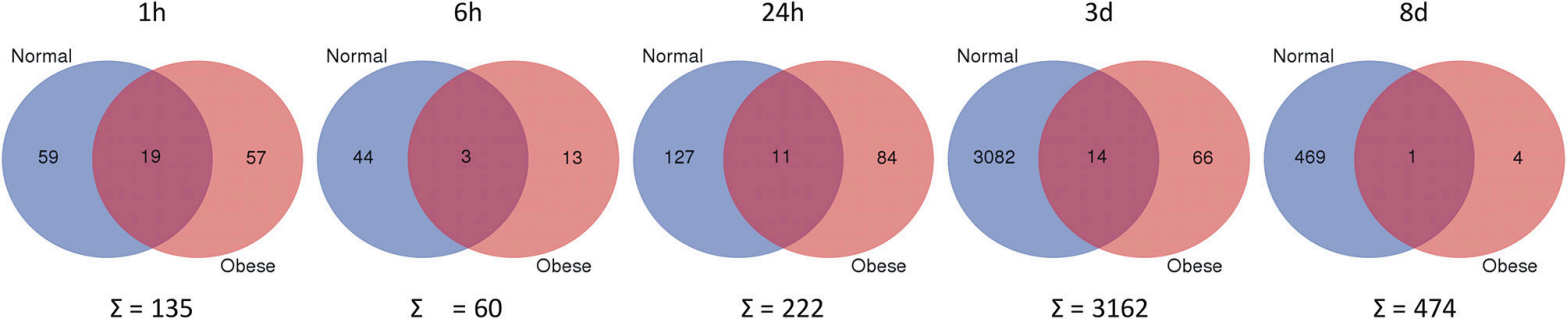
Differentially expressed genes, when comparing trauma vs. control (*p* ≤ 0.05; fold change ≥ ± 1.5) in normal weight and obese mice. Unique and shared genes with their total amount for each time point are shown. Blue = normal; Red = obese. Webtool from Ghent University Bioinformatics and Evolutionary Genomics.

To assess the affiliation of genes to either the normal weight or obese group, the webtool from Ghent University Bioinformatics & Evolutionary Genomics to compare two groups (provided by VIB Ghent University, available at http://bioinformatics.psb.ugent.be/webtools/Venn/) was used for the preparation of Venn diagrams. The gene data analysis revealed that the total amount of DEG was generally higher in normal weight mice at all-time points, whereas obese animals showed less activity (Figure 5). Subgrouping of all genes revealed unexpectedly that only a few genes were shared between both models. Thus, obese animals lack a regular response to the blunt muscle injury of the *extensor iliotibialis anticus*.

### Time dependency of the gene expression profile after muscle trauma in obese vs. normal weight mice reflected by the time lapse heatmap

Due to the complexity of our study design, a comparative time lapse heatmap was generated to normalize trauma to control animals for each time point in their corresponding diet. Consequently, expression levels of genes with at least three significant hits over time regardless of diet were summarized. Eighty-one DEG within these criteria had a stronger impact over the evaluated time frame. Especially 3 days post-injury, the analysis indicated the greatest influence on expression levels regarding regeneration in normal weight mice (Table 3).

**Table 3:** Gene expression levels in fold change values determined by microarray analysis with at least three hits and a fold change ≥±1.5 over all time points independent of diet.

**ID**	**Name**	**Trauma vs. Control**									
		**Normal**					**Obese**				
		**1 h**	**6 h**	**24 h**	**3 days**	**8 days**	**1 h**	**6 h**	**24 h**	**3 days**	**8 days**
107765	*Ankrd1*	1.60	14.26	−**5.81**	**50.36**	13.48	**2.69**	4.38	−2.79	1.15	1.91
11820	*App*	1.30	1.18	−**1.59**	**2.17**	**1.68**	1.06	−1.08	−1.22	1.33	1.18
11829	*Aqp4*	−1.32	−1.20	1.74	−**5.67**	−**2.48**	−1.06	−1.03	**1.66**	−1.79	−1.57
12051	*Bcl3*	**1.92**	1.85	1.21	**2.69**	1.41	**2.31**	1.76	1.23	1.23	−1.06
12226	*Btg1*	1.66	1.42	−1.14	**1.89**	**1.53**	**1.67**	1.56	−1.18	1.33	1.29
12267	*C3ar1*	**2.01**	1.79	2.14	**32.56**	**4.19**	1.30	1.96	**3.03**	5.98	1.72
12363	*Casp4*	**1.63**	2.00	1.31	**2.57**	1.26	1.71	**1.74**	1.37	1.36	1.20
20305	*Ccl6*	**1.84**	**2.35**	2.72	**11.06**	2.56	−1.02	1.46	2.99	3.01	1.39
20307	*Ccl8*	1.11	2.83	**6.35**	**13.98**	**2.75**	−1.73	1.67	5.24	4.12	1.74
20308	*Ccl9*	**2.07**	**3.63**	2.38	**10.02**	2.78	1.00	**1.98**	2.97	3.10	1.19
23833	*Cd52*	1.80	2.14	2.15	**14.45**	2.95	**1.64**	1.45	**2.85**	2.69	2.13
12522	*Cd83*	**2.27**	1.08	−1.15	**1.89**	1.52	**1.91**	1.16	1.11	1.24	1.06
14962	*Cfb*	1.22	1.43	2.53	**3.39**	**1.59**	−1.95	1.34	**2.56**	2.90	1.64
76722	*Ckmt2*	−1.02	−1.00	−**1.63**	−**2.45**	−1.38	1.12	−1.03	−1.51	−**1.63**	1.15
29876	*Clic4*	1.46	**1.62**	−1.06	**2.78**	**1.43**	1.28	1.73	1.10	1.19	1.20
12825	*Col3a1*	**1.53**	1.14	1.66	**4.59**	**3.71**	−1.26	1.04	1.38	2.65	1.48
72042	*Cotl1*	1.28	1.15	1.43	**4.49**	**1.70**	1.16	1.14	**1.53**	1.63	1.29
14219	*Ctgf*	**2.35**	2.35	−**1.96**	**3.70**	1.36	1.91	1.92	−1.15	−1.15	−1.09
13040	*Ctss*	**1.96**	1.80	2.11	**32.07**	4.17	−1.10	1.48	**3.21**	6.13	3.59
16007	*Cyr61*	2.47	1.91	−**2.06**	**2.11**	1.35	**2.90**	1.97	−1.55	−1.30	1.05
72318	*Cyth4*	1.35	1.42	1.37	**6.71**	**1.96**	1.08	1.27	**1.67**	2.18	1.27
13730	*Emp1*	**2.39**	**2.52**	−1.23	**6.12**	1.85	1.86	2.42	1.24	1.95	1.17
74155	*Errfi1*	**2.29**	2.05	−1.32	**2.17**	1.58	**2.12**	1.71	1.09	−1.25	−1.01
14130	*Fcgr2b*	**1.63**	**1.72**	1.71	**11.10**	2.43	−1.23	1.35	1.81	2.49	1.37
14190	*Fgl2*	**2.46**	**2.98**	−1.09	2.86	1.05	**2.76**	3.20	1.17	1.23	1.47
14268	*Fn1*	1.82	1.35	1.00	**6.07**	**3.39**	−1.11	1.16	1.04	1.18	**1.51**
14545	*Gdap1*	−1.10	−1.31	−**1.56**	−**2.37**	−**1.68**	−1.03	−1.20	1.01	−1.31	1.16
14955	*H19*	−1.08	−1.06	−**1.54**	**2.05**	**2.71**	1.18	1.17	−1.82	−1.47	1.53
15430	*Hoxd10*	−2.33	−1.07	**5.59**	−5.67	−2.77	−1.94	−1.24	**3.77**	**1.95**	−2.00
319415	*Hs3st5*	−1.09	−1.44	1.01	−**2.53**	−**1.94**	1.07	−**1.51**	−1.14	−1.06	−1.17
15894	*Icam1*	1.42	1.42	1.04	**1.61**	**1.31**	**1.62**	1.33	1.09	1.26	1.10
15959	*Ifit3*	−1.30	1.64	1.59	**5.29**	1.81	−**1.50**	1.52	**2.99**	1.61	1.90
80719	*Igsf6*	**1.63**	1.18	1.29	**13.78**	2.35	**1.88**	1.68	1.32	2.06	1.49
73914	*Irak3*	**1.65**	**1.58**	1.09	**1.92**	1.38	1.24	1.41	1.08	1.18	1.15
16402	*Itga5*	**2.15**	2.35	1.11	**5.35**	1.75	**2.11**	2.54	1.09	1.35	−1.08
16414	*Itgb2*	**2.03**	2.14	1.43	**9.36**	1.95	**1.60**	1.80	**1.50**	3.19	1.64
277396	*Klhl23*	1.14	−1.16	−**1.56**	−**1.64**	−1.18	1.02	−1.10	−1.22	−**1.70**	−1.10
244864	*Layn*	1.02	**1.60**	1.13	**3.13**	**1.63**	1.01	1.44	1.17	1.53	1.09
18826	*Lcp1*	**2.36**	2.10	1.56	**14.25**	2.97	**2.03**	1.94	**2.01**	3.93	2.00
16822	*Lcp2*	**1.90**	1.91	1.47	**5.60**	1.88	**1.61**	1.76	1.45	2.04	1.29
16854	*Lgals3*	1.42	1.95	1.82	**19.07**	**3.85**	−1.11	1.49	**2.76**	5.87	1.34
56722	*Litaf*	**1.55**	1.94	1.06	**3.73**	1.31	**1.98**	1.85	1.34	1.53	1.11
16948	*Lox*	**2.53**	3.12	−1.14	**12.02**	**2.36**	1.34	2.17	2.06	3.64	1.22
16979	*Lrrn1*	−1.38	−1.44	−**3.01**	**5.06**	**2.49**	1.90	1.20	−1.13	−2.47	1.05
26410	*Map3k8*	**1.69**	2.59	1.09	**1.74**	**1.18**	2.32	2.23	1.04	1.19	1.01
59090	*Midn*	**1.70**	2.87	1.01	**2.70**	**1.41**	2.74	1.74	−1.21	−1.07	1.10
17476	*Mpeg1*	2.10	1.76	1.61	**31.46**	**5.10**	1.33	2.63	**2.54**	6.09	3.55
73656	*Ms4a6c*	**2.01**	2.24	2.85	**29.44**	3.42	−1.08	2.27	**5.73**	5.00	2.26
74843	*Mss51*	1.75	1.94	−**5.13**	**3.62**	**3.14**	1.28	1.07	1.02	1.14	1.87
17748	*Mt1*	1.96	**3.50**	3.75	**6.21**	**2.08**	**3.66**	1.63	2.57	1.90	−1.05
18198	*Musk*	−1.20	1.10	1.70	**3.69**	2.99	1.06	1.39	**1.68**	**1.60**	−1.15
17874	*Myd88*	1.49	3.81	1.36	**4.14**	**1.53**	**2.41**	2.20	1.12	1.41	1.20
228785	*Mylk2*	1.13	1.10	−**1.53**	−**2.16**	−**1.43**	1.04	1.07	−1.36	−1.00	1.21
17937	*Nab2*	1.13	1.77	1.11	**2.28**	**1.49**	**1.66**	2.05	1.18	1.07	−1.07
105855	*Nckap1l*	**1.63**	1.36	1.22	**12.33**	2.06	1.15	1.47	**1.62**	2.89	1.26
18414	*Osmr*	**1.57**	**2.13**	1.09	**2.89**	1.22	1.41	2.08	1.27	1.21	1.04
67041	*Oxct1*	1.03	−1.12	−**1.59**	−**2.25**	−1.55	1.10	−1.02	−**1.58**	−1.46	1.10
170768	*Pfkfb3*	**1.51**	1.20	1.11	−**3.78**	−2.43	−1.11	1.45	−1.21	**1.75**	−1.11
66681	*Pgm1*	**1.57**	1.81	1.09	**3.22**	1.19	**1.91**	1.60	1.06	1.08	1.15
83490	*Pik3ap1*	1.26	1.12	1.15	**4.09**	**1.57**	1.04	1.31	**1.68**	2.27	1.15
234779	*Plcg2*	1.21	1.09	1.11	**2.09**	**1.22**	−1.08	1.13	1.31	**1.53**	1.18
67448	*Plxdc2*	1.39	−1.14	−**1.72**	**1.77**	**1.71**	1.11	−1.18	−1.42	1.06	1.28
243382	*Ppm1k*	1.16	−1.22	−**1.60**	−**2.35**	−**1.80**	1.02	−1.18	−1.46	−1.63	1.10
19264	*Ptprc*	**2.73**	1.59	1.29	**8.35**	**1.95**	**1.83**	1.70	1.67	2.19	1.93
67442	*Retsat*	−1.12	−1.27	−**1.52**	−**1.78**	−1.33	1.09	−1.14	−**1.77**	−1.12	−1.08
74194	*Rnd3*	**1.84**	1.32	−1.13	**1.82**	**1.51**	1.50	1.62	1.09	1.07	1.05
672511	*Rnf213*	1.11	1.54	1.60	**4.13**	**1.44**	−1.10	1.59	**1.95**	2.03	1.29
20194	*S100a10*	**1.84**	**1.56**	1.02	**5.65**	1.61	1.31	1.42	1.62	1.92	1.37
20970	*Sdc3*	1.02	1.24	1.62	**6.20**	**1.57**	−1.05	1.02	**1.81**	2.31	1.30
20345	*Selplg*	**1.50**	1.39	1.28	**6.13**	2.17	**1.54**	1.26	1.60	2.64	1.35
18787	*Serpine1*	**4.44**	3.18	1.18	**9.62**	1.19	**3.69**	3.30	1.19	1.74	1.06
100217426	*Snord49b*	−1.01	1.07	1.46	**2.35**	**1.83**	1.01	−1.31	**1.89**	1.59	1.14
234214	*Sorbs2*	1.07	1.12	1.09	−**2.63**	−**1.98**	−1.02	1.14	−1.07	**1.52**	−1.07
98267	*Stk17b*	1.68	1.23	−1.16	**4.69**	**1.58**	**1.66**	1.08	−1.14	1.14	1.18
21825	*Thbs1*	**4.19**	4.58	−**2.00**	**10.10**	1.63	3.99	2.50	1.20	2.69	−1.22
279572	*Tlr13*	1.36	1.89	1.20	**10.16**	**1.55**	1.33	2.49	**1.70**	3.03	1.14
21923	*Tnc*	1.81	2.79	−**1.64**	**22.69**	**2.44**	1.36	1.97	1.01	2.52	−1.01
21933	*Tnfrsf10b*	1.31	1.39	1.24	**2.05**	1.07	**1.63**	**1.62**	1.10	1.11	1.03
69480	*Ttc9*	1.03	1.52	−**1.66**	**6.89**	**6.18**	1.21	1.90	−1.19	−1.42	1.28
22235	*Ugdh*	**3.18**	**1.56**	1.03	**2.85**	1.58	2.32	1.58	1.02	1.26	1.11
381066	*Zfp948*	**1.57**	1.28	1.03	**1.93**	1.34	**1.76**	1.92	1.07	1.15	1.06

*Red = significantly upregulated, green = significantly downregulated. Significance level is p ≤ 0.05. Log2 values were converted to fold change, and subsequently if value < 1 inverted to get negative values, thus baseline = ± 1. For corresponding significance levels, see Supplementary Table 3*.

### Gene expression for leukocyte migration is central after muscle trauma

**Figure 6:**
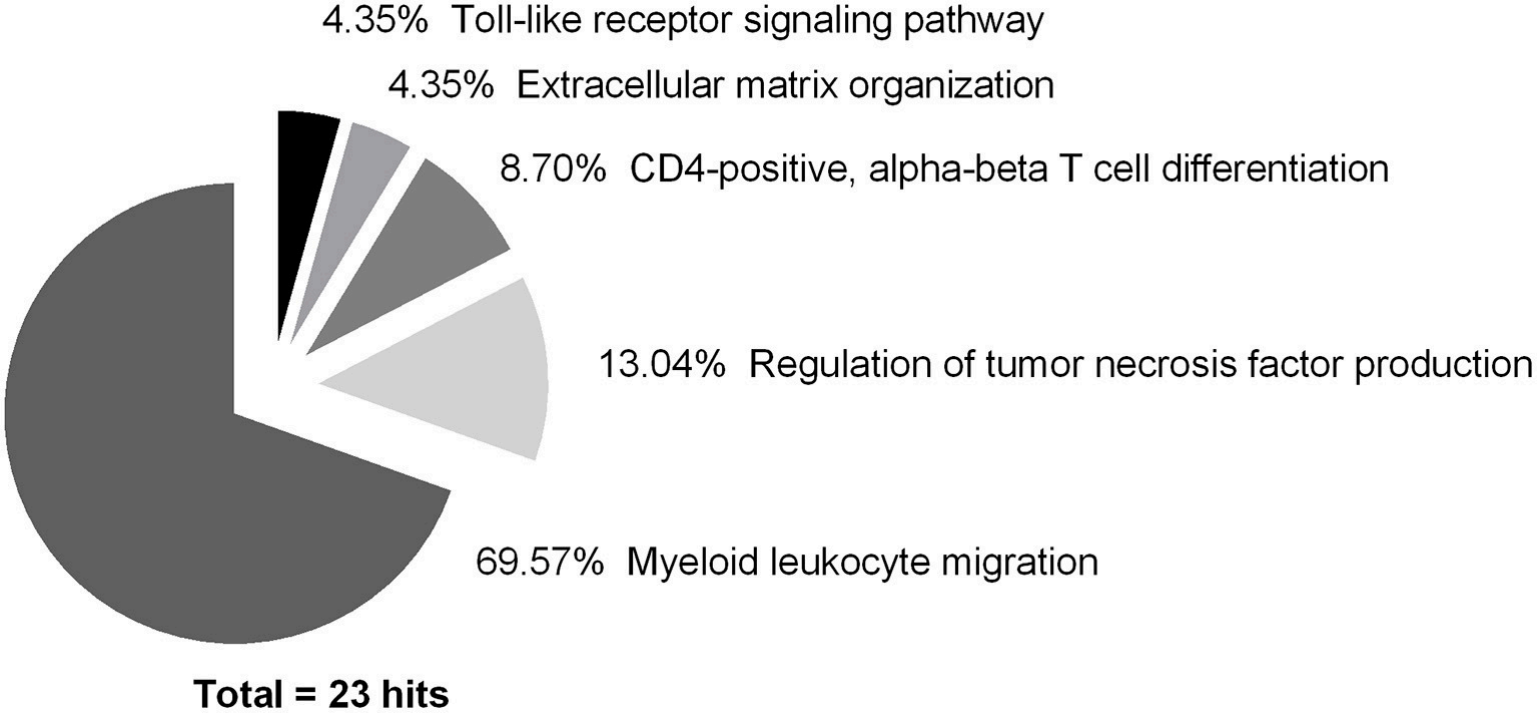
Pathway analysis of differentially expressed genes showing the highest impact over the whole course of time (hits ≥ 3; 23 in total). Data was analyzed using ClueGO (Bindea et al., 2009), a Cytoscape (Lotia et al., 2013) plugin designed by Bindea et al. (2009). Each association to an ontology was counted as one hit, regardless of gene quantity.

All 81 DEG were used to perform a pathway analysis in order to determine their affiliation with GO terms. Some terms were grouped due to their association to a common superordinate term. Five terms in total were identified, namely myeloid leukocyte migration (16 hits; 69.57%), regulation of tumor necrosis factor production (3 hits; 13.04%), CD4-positive, alpha-beta T cell differentiation (2 hits; 8.70%), ECM organization (1 hit; 4.35%), and toll-like receptor (TLR) signaling pathway (1 hit; 4.35%) with high confidence and at least 3 matches to the database (Figure 6).

As the grouping of GO terms was necessary, the underlying captions were grouped into the next superordinate one (Table 4), which was due for CD4-positive, alpha-beta T cell differentiation, regulation of tumor necrosis factor production, and myeloid leukocyte migration.

**Table 4:** Overview of GO terms found by ClueGO.

**GOID**	**GO term**	**Term PValue**	**Associated genes found**
GO:0002224	*Toll-like receptor signaling pathway*	240.0E-6	*Irak3, Myd88, Pik3ap1, Tlr13*
GO:0030198	*Extracellular matrix organization*	76.0E-9	*App, Bcl3, Col3a1, Ctgf, Ctss, Cyr61, Lcp1, Lgals3, Lox*
GO:0032653	Regulation of interleukin-10 production	500.0E-6	*Bcl3, Cd83, Fcgr2b*
GO:0043367	*CD4-positive, alpha-beta T cell differentiation*	1.4E-3	*Bcl3, Cd83, Nckap1l*
GO:0032680	*Regulation of tumor necrosis factor production*	44.0E-6	*Bcl3, Errfi1, Irak3, Myd88, Thbs1*
GO:0032720	Negative regulation of tumor necrosis factor production	500.0E-6	*Bcl3, Errfi1, Irak3*
GO:0042534	Regulation of tumor necrosis factor biosynthetic process	50.0E-6	*Bcl3, Errfi1, Thbs1*
GO:0002685	Regulation of leukocyte migration	1.7E-6	*C3ar1, Icam1, Lgals3, Myd88, Nckap1l, Serpine1, Thbs1*
GO:0070555	Response to interleukin-1	180.0E-9	*Ankrd1, Ccl6, Ccl8, Ccl9, Icam1, Irak3, Myd88*
GO:0030595	Leukocyte chemotaxis	18.0E-9	*C3ar1, Ccl6, Ccl8, Ccl9, Itgb2, Lgals3, Nckap1l, Serpine1, Thbs1*
GO:0071674	Mononuclear cell migration	6.4E-9	*C3ar1, Ccl6, Ccl8, Ccl9, Lgals3, Serpine1, Thbs1*
GO:0097529	*Myeloid leukocyte migration*	340.0E-12	*C3ar1, Ccl6, Ccl8, Ccl9, Itgb2, Lgals3, Myd88, Nckap1l, Serpine1, Thbs1*
GO:0071346	Cellular response to interferon-gamma	8.3E-6	*Aqp4, Ccl6, Ccl8, Ccl9, Icam1*
GO:0071675	Regulation of mononuclear cell migration	14.0E-6	*C3ar1, Lgals3, Serpine1, Thbs1*
GO:0002548	Monocyte chemotaxis	970.0E-9	*Ccl6, Ccl8, Ccl9, Lgals3, Serpine1*
GO:0048247	Lymphocyte chemotaxis	720.0E-6	*Ccl6, Ccl8, Ccl9*
GO:0002690	Positive regulation of leukocyte chemotaxis	190.0E-6	*C3ar1, Nckap1l, Serpine1, Thbs1*
GO:0008625	Extrinsic apoptotic signaling pathway via death domain receptors	8.3E-6	*Icam1, Lgals3, Serpine1, Thbs1, Tnfrsf10b*
GO:0048246	Macrophage chemotaxis	320.0E-6	*C3ar1, Lgals3, Thbs1*
GO:1902622	Regulation of neutrophil migration	250.0E-6	*C3ar1, Myd88, Nckap1l*
GO:0071622	Regulation of granulocyte chemotaxis	600.0E-6	*C3ar1, Nckap1l, Thbs1*
GO:2000351	Regulation of endothelial cell apoptotic process	440.0E-6	*Icam1, Serpine1, Thbs1*
GO:0045766	Positive regulation of angiogenesis	4.6E-6	*Btg1, C3ar1, Itga5, Itgb2, Serpine1, Thbs1*

*Ontology source used was Biological Process. Dotted lines indicate group association. Superordinate terms are underlined. Term PValue is the confidence of affiliation of the result*.

### Differential gene expression profiles after muscle trauma between obese and normal weight mice

The pathway analysis revealed several genes of interest, among them *Ankrd1, App, Aqp4, Bcl3, Btg1, C3ar1, Ccl6, Ccl8, Ccl9, Cd83, Col3a1, Ctgf, Ctss, Cyr61, Errfi1, Fcgr2b, Icam1, Irak3, Itga5, Itgb2, Lcp1, Lgals3, Lox, Myd88, Nckap1l, Pik3ap1, Serpine1, Thbs1*, and *Tlr13*. These genes were evaluated to detect differences in expression levels between the comprised data of normal weight and obese mice (Table 3).

Evaluation of the time lapse heatmap data indicated that several genes are associated with the TLR signaling pathway, namely *Irak3, Myd88, Pik3ap1*, and *Tlr13*. While these genes show upregulation in muscle tissue from normal weight mice (except *Irak3*), obese animals show a comparably lower response to injury. *Myd88* is strikingly upregulated 6 h and 3 days post-injury in normal weight mice. *Tlr13* is not involved in the early response to injury. However, it shows a considerable increase 3 days after trauma in the injured muscle of normal weight animals. In summary, TLR signaling is regularly functioning in normal weight animals while obese mice are hampered in response. ECM is influenced by *App, Bcl3, Col3a1, Ctgf*, *Ctss, Cyr61, Lcp1, Lgals3*, and *Lox*, which were at least partially changed. Overall, both groups show a similar behavior of these genes at all-time points with an exception at day 3. Here, normal weight mice have a more or less markedly increase in gene expression levels, up to 30-fold (*Ctss*), which is indicative that ECM organization was impaired in obese animals due to their overall lack in response. CD4-positive, alpha-beta T cell differentiation is mediated by *Bcl3, Cd83, Nckap1l*, and shows a similar reduction in obese mice. Responses in normal weight animals are stronger when compared to obese mice. Whereas, *Bcl3* and *Cd83* do not show a strong reaction upon injury in general, *Nckap1l* is increased 12-fold 3 days post-injury. Thus, CD4-positive T cell differentiation seems to be mostly impaired by a lack of *Nckap1l* response. *Bcl3, Errfi1, Irak3, Myd88*, and *Thbs1* are involved in the regulation of tumor necrosis factor production and show similar curvatures for normal weight and obese animals, with the exception of 3 days post-injury and *Myd88* in general. Overall, activity is also higher in normal weight animals, suggesting a dysfunctional TNF production cycle in obese individuals. The GO term myeloid leukocyte migration includes the following gene associations: *Ankrd1, Aqp4, Btg1, C3ar1, Ccl6, Ccl8, Ccl9, Icam1, Irak3, Itga5, Itgb2, Lgals3, Myd88, Nckap1l, Serpine1, Thbs1*, and *Tnfrsf10b*. In general, data from injured and non-injured muscle tissue from normal weight and obese mice showed similar curvatures, while three days usually marks a major change for obese animals due to a lack of response. Most genes (*Ankrd1, C3ar1, Ccl6, Ccl8, Ccl9, Itga5, Itgb2, Lgals3, Myd88, Nckap1l, Serpine1*, and *Thbs1*) show a strong increase in gene expression at this time point, making it a vital variable for the orchestration of leukocyte migration to the site of injury. Although obese animals do show a response to injury in some chemokines (*Ccl6, Ccl8*, and *Ccl9*), integrin (*Itgb2*), and galectin (*Lgals3*), a proper function in terms of mediating their effects to attract and bind immune cells is impaired.

**Figure 7:**
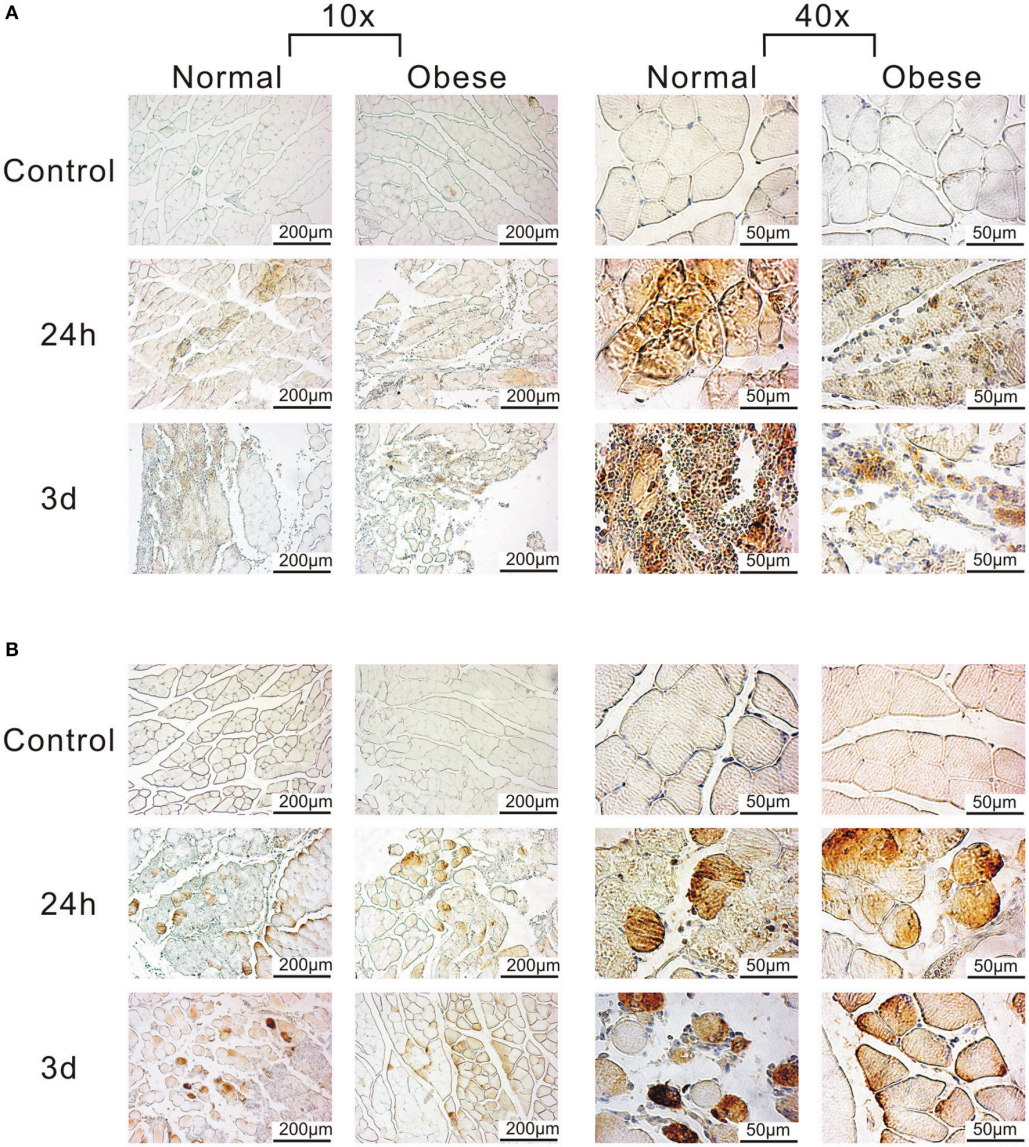
Detection of C3ar and C5ar in muscle tissue of normal weight and obese mice by IHC. **(A)** Anti-C3ar-labeled muscle shows stronger signals for normal weight when compared to obese. **(B)** Anti-C5ar-labeled muscle shows a partially increased signal, and slightly increases over time, also indicating the negative role of obesity. Pictures were taken with Olympus IX81 using Xcellence v.1.2. Scale = 200 μm at 10x magnification; Scale = 50 μm at 40x magnification.

Due to the presence of several complement-related genes within our 81 DEG with major impact on muscle regeneration, we also focused on corresponding factors related to this pathway (Table 5). Here, we found *C1, C3*, and *C5*-associated genes as well as *Cfb* and *F13a1* to be differentially expressed. The complement system is involved in the clearance of tissue debris among other and shows a limited response for obese mice when compared to the normal weight group. It is still active especially after 3 and 8 days post-injury, while the alternative (Cfb) and classical pathway (*C1*) are switched on. IHC analyses of *C3ar* and *C5ar* confirmed a stronger impact of *C3ar* in normal weight animals when compared to obese (Figure 7A), with 3 days showing the highest intensity during muscle regeneration, localized in the damaged area within the muscle. Similarly, despite exhibiting lower expression levels, *C5ar* showed a partially increased signal in the damaged muscle tissue (Figure 7B). Here, intensity levels slightly increase between 24 h and 3 days in both groups, also indicating that obesity hampers the C5ar expression.

**Table 5:** Gene expression levels in fold change values determined by microarray analysis for complement, Wnt and satellite cells.

**ID**	**Name**	**Trauma vs. Control**									
		**Normal**					**Obese**				
		**1 h**	**6 h**	**24 h**	**3 days**	**8 days**	**1 h**	**6 h**	**24 h**	**3 days**	**8 days**
**COMPLEMENT**											
12259	*C1qa*	1.30	1.15	**1.41**	**10.23**	2.43	−1.25	−1.12	1.33	2.22	1.34
12260	*C1qb*	1.33	1.38	**2.35**	**15.07**	4.57	−1.08	−1.00	2.01	2.54	1.56
12262	*C1qc*	1.17	1.12	**1.60**	**11.49**	**3.43**	−1.21	−1.13	1.51	2.04	1.45
81799	*C1qtnf3*	1.09	1.25	−1.06	**19.10**	6.09	1.09	−1.06	1.05	1.85	1.15
12267	*C3ar1*	**2.01**	1.79	2.14	**32.56**	**4.19**	1.30	1.96	**3.03**	5.98	1.72
12273	*C5ar1*	1.59	1.97	1.20	**7.53**	1.59	**1.94**	1.49	**1.35**	2.56	−1.02
14962	*Cfb*	1.22	1.43	2.53	**3.39**	**1.59**	−1.95	1.34	**2.56**	2.90	1.64
74145	*F13a1*	1.65	1.34	1.64	**5.00**	2.03	−1.21	1.33	1.55	1.83	1.04
**WNT**											
12505	*Cd44*	2.24	2.88	1.12	**6.43**	**2.06**	1.80	3.20	1.20	1.88	**1.23**
12385	*Ctnna1*	1.15	1.06	−1.05	**1.87**	**1.23**	−1.02	1.19	−1.12	1.18	1.03
216033	*Ctnna3*	−1.05	−1.05	1.28	−**2.01**	−**1.58**	−1.13	−1.04	1.01	1.21	−1.05
67087	*Ctnnbip1*	−1.25	−1.17	1.28	−**1.52**	−1.29	−1.17	−1.04	1.12	1.06	−1.26
54601	*Foxo4*	−1.02	−1.08	−1.09	−**2.02**	−**1.65**	−1.08	−1.09	−1.05	−1.01	−1.02
26410	*Map3k8*	**1.69**	2.59	1.09	**1.74**	**1.18**	2.32	2.23	1.04	1.19	1.01
17869	*Myc*	**2.58**	2.67	2.06	2.66	1.30	**3.62**	2.27	1.38	1.04	−1.36
20377	*Sfrp1*	−1.02	−1.07	1.24	**3.60**	**2.56**	−1.44	1.13	1.36	2.11	1.05
20319	*Sfrp2*	−1.05	1.05	1.10	**4.08**	**3.02**	−1.18	−1.22	1.40	1.46	1.28
20583	*Snai2*	−1.01	−1.03	−1.01	**2.65**	1.35	−1.21	−1.02	−1.05	1.05	1.03
21406	*Tcf12*	1.03	1.05	−1.12	**1.80**	**1.25**	1.03	1.07	−1.03	−1.05	−1.01
21803	*Tgfb1*	1.19	1.52	1.30	**5.10**	3.23	1.18	1.40	**1.26**	1.86	1.11
21804	*Tgfb1i1*	1.31	−1.00	1.15	**2.39**	1.30	1.26	1.14	−1.09	1.09	1.03
21810	*Tgfbi*	1.19	1.28	−1.13	**3.78**	**1.52**	−1.14	1.15	1.52	1.34	1.06
21812	*Tgfbr1*	1.03	1.11	−1.27	**1.50**	1.30	1.10	1.13	−1.05	−1.00	1.08
21813	*Tgfbr2*	1.22	1.29	−1.08	**2.25**	**1.66**	1.01	1.23	1.05	1.22	1.02
22417	*Wnt4*	−1.14	−1.01	**1.19**	−**1.73**	−**1.51**	−1.12	−1.17	**1.16**	1.04	−1.18
22418	*Wnt5a*	−1.01	−**1.51**	1.02	−**1.35**	−1.16	−1.09	−1.08	−1.28	−1.01	−1.09
**SATELLITE CELLS**											
12490	*Cd34*	1.24	−1.07	−1.17	**2.02**	1.54	−1.03	−1.07	−1.07	1.05	1.09
12552	*Cdh11*	1.13	1.07	−1.25	**2.24**	**2.17**	1.05	1.03	−1.15	1.13	1.12
12555	*Cdh15*	−1.08	−1.04	1.00	**2.18**	1.08	1.03	−1.05	1.00	1.03	−1.08
12558	*Cdh2*	−1.04	−1.03	−1.00	**8.15**	1.31	−1.00	1.09	1.02	1.10	−1.09
12767	*Cxcr4*	1.51	1.09	−1.07	**4.62**	1.51	1.96	1.09	−1.22	−1.05	−1.05
16402	*Itga5*	**2.15**	2.35	1.11	**5.35**	1.75	**2.11**	2.54	1.09	1.35	−1.08
16404	*Itga7*	−1.01	1.18	1.08	**3.73**	**1.22**	1.04	1.05	1.01	**1.24**	−1.04
16409	*Itgam*	**2.17**	2.69	1.78	**13.44**	1.76	1.52	2.03	2.30	2.53	1.42
16410	*Itgav*	1.10	1.06	−1.08	**1.67**	**1.24**	1.03	1.12	−1.08	1.18	**1.15**
16412	*Itgb1*	1.27	1.26	−**1.33**	**2.32**	**1.27**	1.20	1.39	−1.25	1.17	1.20
16414	*Itgb2*	**2.03**	2.14	1.43	**9.36**	1.95	**1.60**	1.80	**1.50**	3.19	1.64
16419	*Itgb5*	1.09	1.11	−**1.50**	**2.19**	1.25	1.04	−1.03	−1.28	1.13	1.01
17295	*Met*	1.20	1.33	1.01	**1.76**	1.38	1.04	1.27	−1.17	−1.10	1.02
17877	*Myf5*	1.01	1.04	−1.12	**1.79**	**1.23**	−1.13	−1.17	−1.04	−1.19	1.05
17878	*Myf6*	1.09	**1.84**	1.01	2.26	1.75	1.21	1.65	−1.06	1.00	−1.06
228785	*Mylk2*	1.13	1.10	−**1.53**	−**2.16**	−**1.43**	1.04	1.07	−1.36	−1.00	1.21
17927	*Myod1*	−1.03	2.39	−1.11	**3.97**	**2.15**	1.14	2.98	1.09	−1.19	1.11
17928	*Myog*	1.06	1.31	**1.63**	**9.94**	3.47	1.06	1.59	**1.38**	**1.30**	−1.23

*Red = significantly upregulated, green = significantly downregulated. Significance level is p ≤ 0.05. Log2 values were converted to fold change, and subsequently if value < 1 inverted to get negative values, thus baseline = ± 1. Values with at least one significant value ≥ ± 1.5 are shown. For corresponding significance levels, see Supplementary Table 4*.

As several pathways and genes for the clearance of tissue debris and immune response were found, a priority was also made for pathways involved in the proliferation and differentiation of cells. In this regard, we assessed the Wnt-pathway, responsible for the symmetric expansion of satellite cells and is required for the differentiation of its progenitors, and satellite cell-related genes (Table 5). Wnt-related genes (*Cd44, Ctnna1, Ctnna3, Ctnnbip1, Foxo4, Map3k8, Myc, Sfrp1, Sfrp2, Snai2, Tcf12, Tgfb1, Tgfb1i1, Tgfbi, Tgfbr1*, and *Tgfbr2*) were also changed upon injury for normal weight mice, but with no significant changes in the obese group.

Satellite cells are crucial key players in muscle regeneration due to their active role in forming new myofibers (Table 5). Here, *Cd34, Cdh11, Cdh15, Cdh2, Cxcr4, Itga5, Itga7, Itgam, Itgav, Itgb1, Itgb2, Itgb5, Met, Myf5, Myf6, Mylk2, Myod1*, and *Myog* were found to be altered. In general, satellite cell-related genes were changed upon injury in normal weight mice, whereas obese animals clearly lack a distinct response to the same injury impact.

### Differences in satellite cell numbers and validation of gene expression

In order to validate the previous findings, we used IHC and qPCR analysis to affirm the presence of *Pax7*^+^ cells and the expression levels of *Ankrd1, C3ar1, Mpeg1, Ccl8*, and *Myog*.

**Figure 8:**
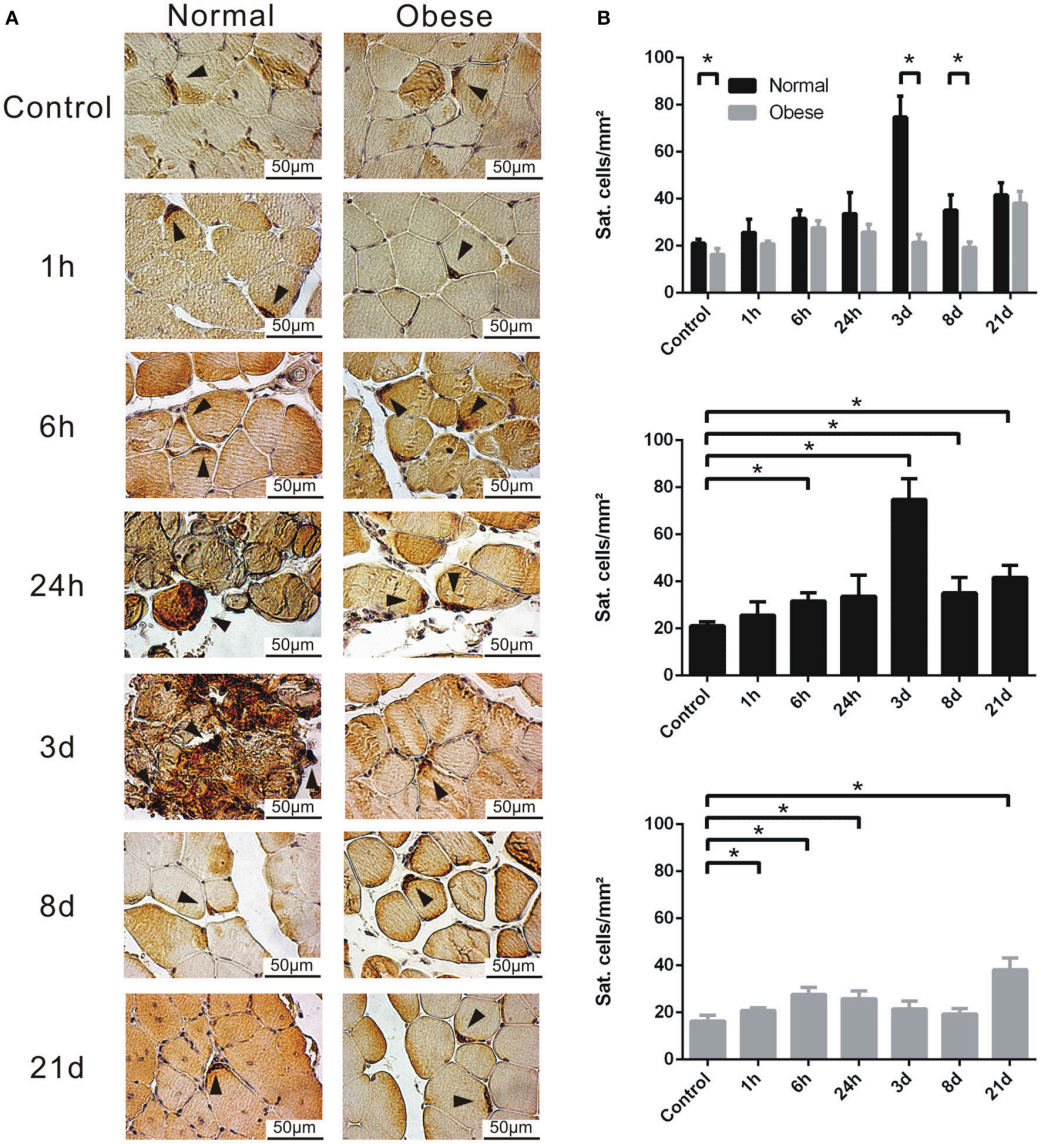
Determination of Pax7-positive satellite cells by IHC. **(A)** Tissue sections of female normal weight and obese C57BL/6J mice, labeled with anti-Pax7, and stained with DAB. Triangle = satellite cell. Pictures were taken with Olympus IX81 using Xcellence v.1.2. Scale = 50 μm at 40x magnification. **(B)** Calculated satellite cells per mm^2^. Six animals per time point à three sections per animal were independently counted by three researchers, averaged and depicted. Black = normal weight; Gray = obese. Statistical analysis by homoscedastic two-sided *t*-test. ^*^Indicates *p* ≤ 0.05.

Upon evaluation of the satellite cell populations within the muscle tissue of sham and injured normal weight and obese C57BL/6J mice in histological stainings (Figure 8A), we observed notable differences. While normal weight animals exhibited higher numbers of satellite cells per mm^2^ altogether within controls, 3 and 8 days showed significantly higher results. Also, 3 days seems to confirm to be a crucial turning point for Pax7-positive cells (Figure 8B). In comparison, obese animals lack this strong increase of satellite cell numbers and stick relatively close to basal levels. Interestingly, both groups showed higher numbers at 21 days, possibly indicating the renewal of the stem cell pool.

**Figure 9:**
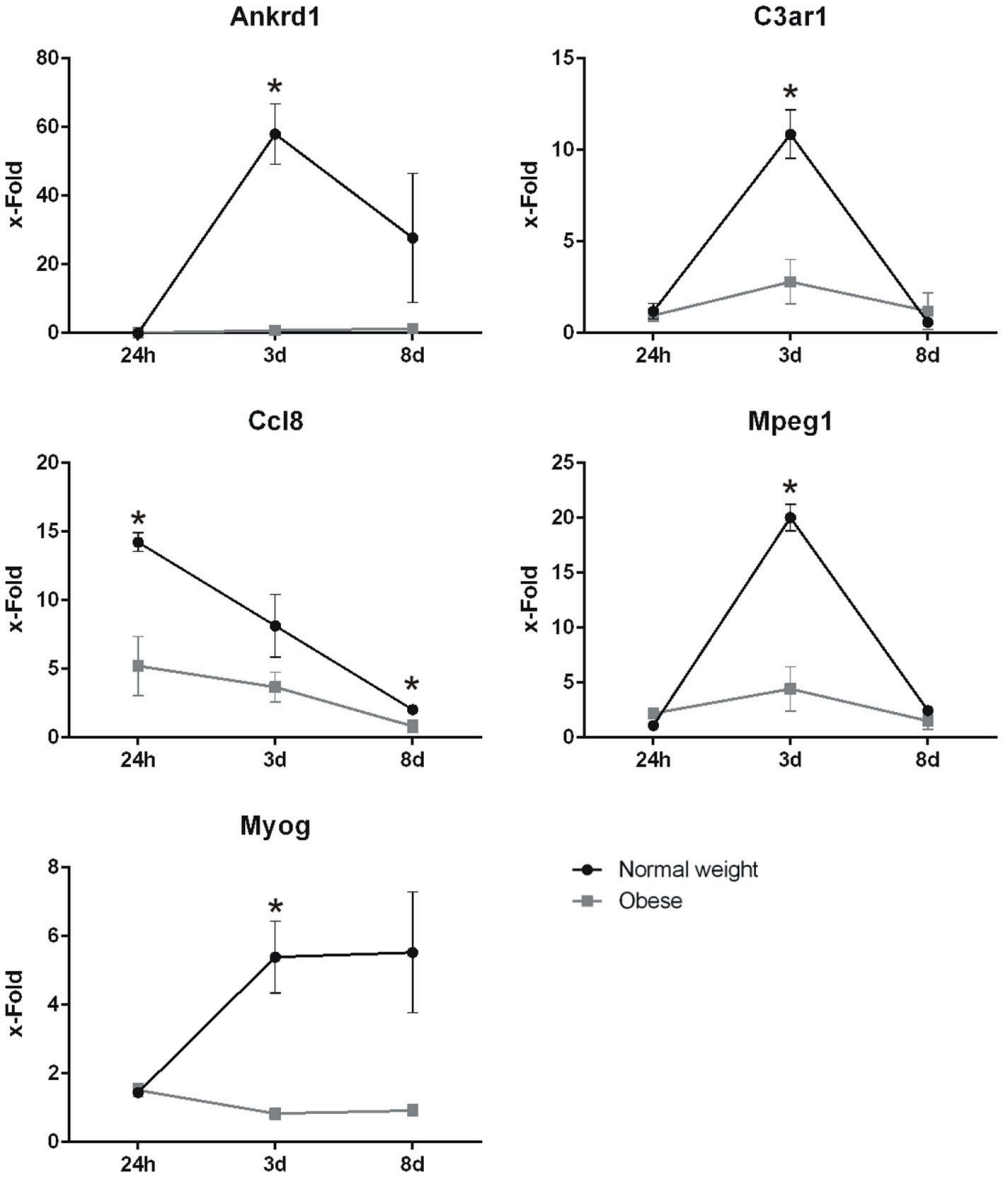
Expression levels of *Ankrd1, C3ar1, Ccl8, Mpeg1*, and *Myog* determined by qPCR on *Ppia* as housekeeper. X-fold values ±SEM were determined by normalization on corresponding controls of each time point and diet. Statistical analysis by two-sided homoscedastic *t*-test. ^*^Indicates *p* ≤ 0.05, *n* = 3.

In order to reaffirm the expression levels of several genes, including *Ankrd1, C3ar1, Mpeg1, Ccl8*, and *Myog*, qPCR were performed. Here, gene expression levels detected in our microarray analysis could be confirmed. Similarly, although upregulated, the endpoint marker of differentiation *Myog* was significantly altered 3 days post-injury, while it shows similar but non-significant levels after day 8 (Figure 9).

## Discussion

Obesity, characterized by dysfunctional and excessive adipose tissue and a chronic low-grade inflammation, is associated with severe co-morbidities that affect cell metabolism (Upadhyay et al., 2018). The accompanied metabolic changes, increased levels of adipocytokines and lipid metabolites seem to impair muscle regeneration by altering the interplay of several cell populations, such as the interaction of satellite cells with MΦ. Consequently, the rebuilding of smaller myofibrils, increased interstitial spaces, higher amounts of collagen deposition, a reduction in the amount, and regenerative ability of satellite cells were observed (Langen et al., 2006; Tilg and Moschen, 2008; Gregor and Hotamisligil, 2011; Ouchi and Walsh, 2012; Adamczak and Wiecek, 2013; Akhmedov and Berdeaux, 2013; Makki et al., 2013; Cao, 2014; D'Souza et al., 2015; Caslin et al., 2016; Hardy et al., 2016; Liu et al., 2016; Chapman et al., 2017; Sinha-Hikim et al., 2017).

In this study, we first analyzed the effects of diet-induced obesity (DIO) on muscle regeneration and fibrosis formation in the skeletal muscle of C57BL/6J mice after injury. Since there is evidence that early lifetime nutrition has an influence on the regeneration ability (Woo et al., 2011), parental mice already received a HFD at least 1 week before breading. DIO resulted in a statistically significant increase in body weight as described earlier (Lai et al., 2014). Consequently, the non-invasive animal model of blunt crush muscle injury in normal weight and DIO mice which is based on a drop tower approach resulted in muscle damage in all mice and was verified by MRI and morphological analyses.

Muscle damage was assessed in the T2-weighted MRI images, in which a clear contrast between the edema in the injured (hyperintense signal) and healthy muscle was observed. In injured muscles of both, normal weight and obese mice, edema and hemorrhage was already seen 1 h after injury, indicated by a high T2W-RARE signal for edema and a low T2W-RARE signal for hemorrhage. Whereas, edema was visible until 3 days post-trauma in the injured muscle tissue of the normal weight mouse, the edema in the injured muscle of the obese mouse is still observed 8 days after injury, pointing to a delay in edema elimination. However, results of other studies revealed that the peak of edema depends on the kind and degree of injury applied through the injury model and is usually visible between 7 h up to 2 days after injury (Mattila et al., 1995; Wishnia et al., 2001). In addition, changes in the T2 signal might not exclusively be due to inflammation, and water and fat content, but might also depend on intrinsic cellular features, among them composition of the cell membrane and its degree of disruption, as well as on disease-induced changes, like the development of a metabolic syndrome (Dunn and Zaim-Wadghiri, 1999; McMillan et al., 2011; Miller and Toth, 2013; Pratt et al., 2013; Acevedo et al., 2017). Since our results did not show statistically significant differences of force input into the muscle tissue of normal and obese mice by our drop-toper based trauma model, it seems that obesity-based physiological changes as well as changes in the composition of the cell membrane of muscle cells of obese mice might contribute to the late T2 peak in obese muscle tissue. This assumption is underlined by our data, which indicate obesity-associated as well as trauma-induced changes in fatty acid composition in muscle tissue of obese mice in comparison to normal weight mice (Werner et al., 2018).

Within the first 24 h after trauma induction, infiltration of immune cells, bloating of myofibers, and necrotic processes were observed. Furthermore, a delay in fluid retention and a large amount of fibrous tissue were observed in skeletal muscle of obese mice. These findings are in line with data obtained from other physical muscle injury models (Akhmedov and Berdeaux, 2013; D'Souza et al., 2015; Hardy et al., 2016). To explain the high degree of fibrosis in obese mice, microarray analysis was performed to detect changes in the expression levels of genes involved in ECM remodeling, among them collagens, fibronectin, elastin, proteoglycans, and laminin. We identified several significant differences in the expression levels of genes involved in ECM organization, among them *App, Bcl3, Col3a1, Ctgf*, *Ctss, Cyr61, Lcp1, Lgals3*, and *Lox*. Obese mice usually showed lower expression levels and lower numbers of ECM-associated cells, a stiffer and excessive ECM with an impaired signaling cascade when compared to normal weight individuals, whereas nutritional cues present in obesity are also of note due to their influence on increased free fatty acids (Garg et al., 2015; Lin et al., 2016; Chapman et al., 2017; Werner et al., 2018). However, as changes in ECM organization occur, infiltration of immune cells and clearance of tissue debris becomes problematic, thus leading to a hampered response in obese individuals (Mann et al., 2011; Akhmedov and Berdeaux, 2013; Urciuolo et al., 2013; Garg et al., 2015; Sziksz et al., 2015; Darby et al., 2016; Lin et al., 2016). Similarly, our analysis also revealed increased amounts of fibrous tissue in the muscle of obese animals. In contrast to the elevated fibrosis, we also observed a down-regulation of ECM-related gene expression levels responsible for degradation, which is in line with lower numbers of cells responsible for ECM organization in obese mice (Akhmedov and Berdeaux, 2013; Urciuolo et al., 2013).

Further detailed analysis of our previous published microarray data (Werner et al., 2018) of skeletal muscle after blunt trauma induction in normal weight and obese C57BL/6J mice over 8 days clearly revealed that normal weight animals showed a significant response to injury, resulting in a peak of alterations in expression levels 3 days post-injury, whereas regeneration in obese animals was impaired, seen in the much lower number of total DEG. Overall, 81 DEG were identified with at least three significant hits in all time points, thus revealing the most striking candidates regarding muscle regeneration. Additionally, it led to the detection of genes with non-significant changes with high values, among them *Ankrd* (6 h and 8 days; normal), *C3ar1* (3 days; obese), *Mpeg1* (3 days; obese), and *Ccl8* (24 h and 3 days; obese).

Pathway analysis revealed the association of our 81 DEG in TLR signaling, ECM organization, CD4-positive, alpha-beta T cell differentiation, regulation of tumor necrosis factor production, and myeloid leukocyte migration with high confidence and at least three matches to the database.

Within the TLR pathway, we identified genes involved in the pro-inflammatory MΦ mediated response (*Myd88, Pik3ap1*), or in weakening the pro-inflammatory response (*Irak3*) (GeneCards, 2017a,b,c). Whereas, *Myd88* showed an immediate response to injury within 6 h post-injury, *Irak3* expression levels were less altered in general. TLRs have been shown to play a major role in innate immunity and are involved in regulating muscle regeneration in response to traumatic injury (Tidball and Villalta, 2010; Tidball et al., 2014). In this context, *TLR2* deficiency resulted in a reduction of the number of MΦ in injured muscles, leading to delayed clearance of necrotic tissue and an impaired reconstruction of muscle structure (Mojumdar et al., 2016). The connection of *TLR4* and lipid metabolism makes *TLR4* a potential target to control adipose tissue inflammation and insulin resistance in obesity (Phieler et al., 2013). Interestingly, *TLR4* gene expression and protein levels seem to be significantly upregulated in T2DM, eventually leading to insulin resistance. This correlates to abnormal *TLR4* expression found in obese individuals, likely caused by increased free fatty acid levels which boost the likelihood of insulin resistance (Könner and Br üning, 2011). Particularly sphingolipid ceramides, derived from saturated fatty acids, are linked to metabolic stress by excessive nutrient availability and inflammatory cytokines to insulin resistance (Summers and Nelson, 2005).

*CD4* is a surface marker for lineage committed T-cells that mature toward specialized features required for cell processes (Burzyn et al., 2013; Yui and Rothenberg, 2014). Four genes (*Bcl3, Cd83, Fcgr2b*, and *Nckap1l*) associated with this term were found and exhibited lower expression levels in muscle tissue of obese mice when compared to those of normal weight mice. Although *CD83* did not show distinct changes between both groups, major alterations were noticeable 3 days post-injury. Especially *Fcgr2b* and *Nckap1l* show a marked increase in normal weight mice, indicating a higher incidence for clearance of debris by immune cells. T-cells play a role of in the development of insulin resistance in obesity (Bornstein et al., 2000; Catalán et al., 2013). They are involved in pro-inflammatory cytokine production of monocytes and MΦ, which remove tissue debris in the initial degenerative phase after injury (Burzyn et al., 2013; Makki et al., 2013).

T-cells, most notably Treg, can prime other immune cells, and also exhibit various functions related to the regulation of body weight, insulin-resistance and glucose tolerance (Sakaguchi et al., 2008; Winer et al., 2009; Catalán et al., 2013; Chapman and Chi, 2017; Cinkajzlová et al., 2017). This subpopulation declines in obesity, while various negative immune cell-related effects arise (Catalán et al., 2013). We detected only little activity within this pathway within our obese model, indicating that obesity exhibits a deregulated T-cell response, which potentially further enhances the aberrant muscle regeneration (Chapman and Chi, 2017; Mauro et al., 2017). However, further studies focusing on this pathway are necessary to conclude our assumptions.

Closely linked to T-cell functions is the regulation of tumor necrosis factor (TNF) production, (AmiGO, 2017). Surprisingly, we did not detect any major effect of injury and obesity on the expression levels of TNF or the associated genes *Bcl3, Errfi1, Irak3, Myd88*, and *Thbs1* in our obese model. Usually, TNF is increased in obese individuals due to chronic low grade inflammation, with a major impact on muscle regeneration following injury due to its effects on inflammatory responses by recruiting and modulating MΦ populations (Gregor and Hotamisligil, 2011; Akhmedov and Berdeaux, 2013; Tateya et al., 2013; Sinha et al., 2017). As T-cells are able to regulate the response of other (TNF-producing) immune cells, a lack in modification and the subsequent recognition and removal of tissue debris results in a delay of muscle regeneration (Akhmedov and Berdeaux, 2013; Catalán et al., 2013; D'Souza et al., 2015; Chapman and Chi, 2017; Sinha et al., 2017). Additionally, *TNF-*α is a pro-inflammatory cytokine that is closely tied to the pathogenesis of obesity and insulin-resistance. Whereas, this pathway seems to be dysfunctional due to lack of activity in our obese model, a compensatory upregulation of other cytokines and co-morbidities were described previously (Jung and Choi, 2014).

We found several genes associated with the pathway myeloid leukocyte migration, namely *Ankrd1, Aqp4, Btg1, C3ar1, Ccl6, Ccl8, Ccl9, Icam1, Irak3, Itga5, Itgb2, Lgals3, Myd88, Nckap1l, Serpine1, Thbs1, Tnfrsf10b*, which are involved in cell-adhesion and immune cell recruitment and retention. These genes exhibit stronger changes in normal weight mice, while our obese animals only showed a limited response. Manicone et al. described several factors of obesity on, but not limited to, (myeloid) leukocyte migration (Manicone et al., 2016). Although they focused on pulmonary immune cells, a correlation to skeletal muscle by predominant populations of M1Φ, leukocytes, and neutrophils is commonly recognized (Tidball and Villalta, 2010; Pillon et al., 2013; Manicone et al., 2016). As T-cells are involved in recruiting, maintaining and altering immune cell populations, a close link can be made between this pathway and CD4-related mechanisms (Catalán et al., 2013; Chapman and Chi, 2017). Notably, *Ankrd1* and *Ccl8* expression levels were confirmed by our qPCR data supporting the involvement of cell-adhesion and immune cell recruitment during the evaluated time points. Macrophage expressed gene 1 (*Mpeg1*) although not listed above, was also strongly increased as shown by our qPCR results most notably 3 days post-injury, derived from the peak inflammatory response of immune cells to trauma.

Another striking feature of our study is the lack of activity in several genes associated to the complement system in our obese model, namely *C1qa, C1qb, C1qc, C1qtnf3, C3ar1, C5ar1, Cfb*, and *F13a1* (see Table 4). *C3aR* and *C5aR* are involved in the development of insulin resistance in adipocytes by MΦ infiltration, while the complement system is generally needed for sensing, opsonization, and removal of tissue debris after trauma (Phieler et al., 2013; Vlaicu et al., 2016). Here, we see both, upregulation of genes in the classical (*C1*) and the alternative complement pathway (*C3*) in response to trauma, notably 3 days post-injury in normal weight mice. In contrast, genes for the classical pathway were not altered in obese animals after trauma, although obesity has been described to lead to an exaggerated activation of the classical pathway (Vlaicu et al., 2016). Although complement activation is not equal to expression levels of genes, we observed a stronger activation of genes in the alternative pathway. Here, we could show that C3ar signal intensity was increased in tissue sections of normal weight animals when compared to obese, as well as the expression levels of *C3ar1* in our qPCR data. Phieler et al. described the involvement of *C5ar* in MΦ accumulation and M1Φ polarization in obese adipose tissue (Phieler et al., 2014). Although *C5ar* expression was barely altered in the muscle tissue of obese animals, enhanced *C5a* levels after trauma may still signal via the remaining *C5ar* on muscle cells (Burk et al., 2012). Similarly as described for C3ar, IHC staining revealed partially increased signal intensities of C5ar in normal weight animals compared to obese animals. As a limitation of the study, the gene screening approach was not extended to the protein and functional level. Summarizing, the removal of tissue debris and the function of the complement system seems to be hampered within our obesity model, making it unable to properly react to the stimulus.

Changes in gene expression were also detected in the Wnt-pathway among them especially frizzled-related genes, which were elevated 3 and 8 days post-injury in normal weight animals only. Whereas, we did not detect major alterations in gene expression levels for the β-catenin destruction complex, we noticed changes in Wnt proteins (*Wnt1-16*). However, none of those genes were upregulated greater than 2-fold, while only two (*Wnt4* and *Wnt5a*) met our criteria of ≥ ± 1.5-fold difference in regulation. This indicates a severe impact on cell maintenance for obese animals, as they showed only little and unspecific signs of Wnt expression. The downstream targets of Wnt *CD44, Myc*, and *Foxo4* were significantly changed, thus promoting Wnt signaling dependent growth and differentiation (Tortelote et al., 2017). Both, obese and normal weight mice show an upregulation of *Myc* as an immediate response to injury, potentially promoting the proliferation of resident cells, while later on, *CD44* is up- and *Foxo4* down-regulated in normal weight mice. Obese animals lack this response in expression. In general, Wnt proteins are commonly associated with the regulation of cell proliferation, differentiation, and migration. β-catenin, a major player within Wnt, is subjected to a permanent turnover by a destruction complex functioning as an “on/off” switch (Guo and Wang, 2009; Tortelote et al., 2017). *Wnt5a* is associated with the non-canonical pathway in Wnt signaling, controlling intracellular calcium levels. Despite recent findings from Tang et al. we did not find any distinct changes in the upregulation of *Wnt5a* which is supposed to correlate with early-onset obesity (Tang et al., 2018). Fuster et al. described *Wnt5a* as a gene closely correlated with obesity-related metabolic dysfunction by promoting insulin resistance via augmentation of tissue inflammation (Fuster et al., 2015). In addition, the Wnt- and TGF-pathways are closely intertwined on several molecular levels. TGF and Wnt reciprocally regulate their ligand production, hence establish a gradient within the extracellular space and also cross-talk within the nucleus (please note: this interaction is not limited to Wnt signaling, but also involves Hh, Notch, IL, and IFNγ) (Guo and Wang, 2009).

Satellite cells are under direct influence of Wnt and recognized as major players in muscle regeneration upon injury. A stimulus such as a mechanical injury recruits neutrophils and activates the stem cells resident in the muscle (Shi and Garry, 2006; Yin et al., 2013; D'Souza et al., 2015). These cells migrate toward the site of injury and start proliferating, partially losing their stem cell character (myoblasts) as they strive toward differentiation to form myotubes. This process can be assessed by several myogenic regulatory factors (MRFs), like *Pax7, Myf5, MyoD*, and *MyoG*. During the differentiation program, myoblasts fuse to form multinucleated myofibers, which bundle together to rebuild healthy and functional muscle tissue (Shi and Garry, 2006; Keire et al., 2013; Yin et al., 2013; Rocheteau et al., 2015). Although this process is much more complex, we assessed two major classes of genes relevant for satellite cells in muscle regeneration: Adherence (cadherins and integrins) and MRFs. We found cadherins to be significantly upregulated 3 days post-injury in normal weight mice. Integrins showed similar behavior, while *Itga5, Itgam*, and *Itgb2* were activated within 1 h post-injury, potentially advocating for the recruitment of immune cells. Interestingly, *Itga5* and *Itgb2* were upregulated within 1 h for obese animals but did not show a response to injury at later time points. *Myod1* and *Myog* were notably altered in normal weight mice. Both genes are known as an endpoint of differentiation (Chargé and Rudnicki, 2004; Kim et al., 2013; Yin et al., 2013; D'Souza et al., 2015). *Myf5*, a marker for early satellite cell commitment toward proliferation, only showed little yet significant upregulation. *Pax7*, the unique marker for quiescent and activated satellite cells, did not show any changes. Although we expected changes within this marker in our model, the expression threshold of *Pax7* was likely lost due to background noise derived from the low cell count in tissue, previously described at 550 sat. cells on 1 mg muscle tissue (Bentzinger et al., 2012). However, to circumvent this issue, we analyzed total satellite cell numbers per mm^2^ in tissue sections by IHC. Here, total numbers were lower in obese mice, showing a significant decrease for control, at 3 and 8 days post-injury when compared to normal weight. Our findings support previously published results, although the total numbers per time point are lower in comparison (Murphy et al., 2011). While comparing different approaches of injuries, Hardy and coworkers showed that a mechanical approach (here: freezing) led to the overall lowest numbers of satellite cells (Hardy et al., 2016). In addition, 3 days post-injury is probably a major tipping point for polarization of M1Φ to M2Φ, likely a link to the promotion of proliferation from M1Φ to the increase in differentiation promoted by M2Φ (Kharraz et al., 2013; Saclier et al., 2013). This notion is supported by the increased expression of *Myod*, and especially *Myog*, the terminal marker of differentiation seen in our microarray and confirmed by qPCR analysis.

Summarizing, we suggest that normal weight animals show a distinct and differentiated response to injury by the interaction of immune cells with satellite cells to start the proliferation and differentiation procedure. In contrast, the obese animals did show some signs of activity within integrins, but almost completely lacked a response for cadherins and MRFs, thus not eliciting the proper recruitment of necessary immune cells and impairing the satellite cell proliferation and differentiation.

## Conclusion

We used a drop tower unit to induce an indirect blunt muscle injury to *extensor iliotibialis anticus* in female normal weight and obese C57BL/6J mice, thereby not finding any significant differences in force input into the muscle between both groups. MRI and HE-staining confirmed morphological changes derived from the injury, while Sirius Red staining and targeted gene expression analysis of our microarray data revealed altered ECM organization and increased fibrosis formation in obese mice. In general, we further assessed genome-wide gene expression by an unbiased analysis of our microarray data (Werner et al., 2018) and confirmed that obesity hampers the regeneration process. Furthermore, our results showed a distinct and differentiated response to injury for normal weight animals, while obese mice had an overall lower amount of total DEG in all time points. We found 81 DEG in both groups to be significantly changed in at least three time points, by using a time-lapse heatmap. Pathway analysis showed these genes to be involved in myeloid leukocyte migration, regulation of tumor necrosis factor production, CD4-positive, alpha-beta T cell differentiation, ECM organization, and TLR signaling with high confidence. Interestingly and somewhat controversial, obese mice, receiving their diet already during prenatal development, showed an overall lack of response in gene expression levels for most genes. In this regard, we also analyzed genes associated with Wnt and satellite cell physiology, thereby showing striking interferences in our obese model. We were able to further confirm the notion, that obesity alters satellite cell numbers and negatively affects the expression levels of *Ankrd1, C3ar1, Ccl8, Mpeg1*, and *Myog*, as seen in our qPCR results. This indicates that obesity influences the response to injury even more severely than assumed previously. Here, we provide new potential targets for further studies that may significantly affect the outcome of muscle regeneration in normal weight and obese individuals.

## Availability of data

Complete microarray data are available at Gene Expression Omnibus (accession number: GSE103726).

## Author contributions

Study design, funding, and supervision by MW and UK. PX, CH, TK, and J-UW performed animal experiments. UK, PX, J-UW, AP, SM, MH-L, LdR, DW, and LD were involved in establishment of the drop-tower device for the induction of a blunt muscle trauma. LdR, DW, and LD designed the force input setup, while J-UW, LdR, DW, and LD performed the experiments for the determination of force input into the muscles of normal weight and obese mice. MRI scans were performed by PX, AA, and VR. Morphological and immunohistochemistry analyses were performed by PX, SM, CH, TK, J-UW, AP, and MH-L. AS, MJ, PG, and J-UW designed and discussed the microarray method and script for evaluation. J-UW evaluated the corresponding data with valuable input from UK, MW, AS, MJ, PG, and MH-L. UK, MW, MH-L, AS, VR, LD, PX, and JW wrote the paper with input from the other authors. All authors read the final manuscript and approved it.

### Conflict of interest statement

The authors declare that the research was conducted in the absence of any commercial or financial relationships that could be construed as a potential conflict of interest.
