# Supplementary material for: Diet-Induced Obesity Affects Muscle Regeneration After Murine Blunt Muscle Trauma—A Broad Spectrum Analysis
Source: Front Physiol. 2018 Jun 5;9:674. doi: 10.3389/fphys.2018.00674 (PMC5996306; doi:10.3389/fphys.2018.00674)
Supplement: Supplementary file 2 [file Table_2.docx]

Diet-induced obesity affects muscle regeneration after murine blunt muscle trauma – a broad spectrum analysis

Pengfei Xu^1†^, Jens-Uwe Werner^1†^, Sebastian Milerski^1^, Carmen Hamp^1^, Tatjana Kuzenko^1^, Markus Jähnert^2^, Pascal Gottmann^2^, Luisa de Roy^3^, Daniela Warnecke^3^, Alireza Abaei^4^, Annette Palmer^5^, Markus Huber-Lang^5^, Lutz Dürselen^3^, Volker Rasche^4^, Annette Schürmann^2^, Martin Wabitsch^6^*, Uwe Knippschild^1^*

* Correspondence: Prof. Dr. Uwe Knippschild, uwe.knippschild@uniklinik-ulm.de and Prof. Dr. Martin Wabitsch, martin.wabitsch@uniklinik-ulm.de

Sup. Tab. 2: P-values and descriptions for fibrosis formation in lean and obese C57BL/6J mice. Red indicates p ≤ 0.05.

|  |  | **Trauma vs. Control** | | | | | | | | | |  |
| --- | --- | --- | --- | --- | --- | --- | --- | --- | --- | --- | --- | --- |
|  |  | **Normal** | | | | | **Obese** | | | | |  |
| **ID** | **Name** | **1h** | **6h** | **24h** | **3d** | **8d** | **1h** | **6h** | **24h** | **3d** | **8d** | **Description** |
| **Pro-fibrotic** | | | | | | |  | | | | |  |
| 11475 | *Acta2* | 5,39E-01 | 5,60E-01 | 6,84E-01 | 6,21E-03 | 5,69E-01 | 7,96E-01 | 6,82E-01 | 6,99E-01 | 4,14E-01 | 4,38E-01 | actin, alpha 2, smooth muscle, aorta |
| 11606 | *Agt* | 4,23E-01 | 4,50E-01 | 1,11E-01 | 1,93E-02 | 1,10E-01 | 1,51E-01 | 7,76E-01 | 8,45E-04 | 7,21E-01 | 5,52E-02 | angiotensinogen (serpin peptidase inhibitor, clade A, member 8) |
| 20302 | *Ccl3* | 1,56E-01 | 4,11E-01 | 1,65E-01 | 1,13E-01 | 6,46E-03 | 4,13E-02 | 2,79E-01 | 2,80E-02 | 2,99E-01 | 2,76E-01 | chemokine (C-C motif) ligand 3 |
| 14219 | *Ctgf* | 9,33E-03 | 1,97E-01 | 1,04E-02 | 4,01E-02 | 2,57E-01 | 1,41E-01 | 2,35E-01 | 4,40E-01 | 2,88E-01 | 7,29E-01 | connective tissue growth factor |
| **Anti-fibrotic** | | | | | | |  | | | | |  |
| 16154 | *Il10ra* | 3,40E-01 | 4,17E-01 | 1,65E-01 | 3,03E-02 | 9,72E-02 | 1,98E-02 | 6,66E-01 | 3,42E-02 | 2,68E-01 | 6,54E-01 | interleukin 10 receptor, alpha |
| 16155 | *Il10rb* | 3,05E-01 | 8,18E-01 | 3,41E-01 | 7,22E-03 | 1,32E-01 | 3,92E-01 | 5,61E-01 | 5,60E-01 | 2,55E-01 | 1,62E-01 | interleukin 10 receptor, beta |
| 16164 | *Il13ra1* | 1,47E-01 | 1,16E-01 | 5,74E-01 | 5,23E-03 | 7,78E-02 | 2,23E-01 | 3,38E-01 | 6,78E-01 | 4,09E-01 | 9,78E-02 | interleukin 13 receptor, alpha 1 |
| **Collagen components** | | | | | | |  | | | | |  |
| 12816 | *Col12a1* | 2,83E-01 | 4,67E-01 | 2,52E-02 | 9,18E-02 | 3,08E-02 | 5,31E-01 | 6,57E-01 | 7,82E-02 | 4,76E-01 | 7,99E-01 | collagen, type XII, alpha 1 |
| 12818 | *Col14a1* | 3,20E-01 | 6,84E-01 | 8,63E-01 | 1,01E-01 | 1,99E-03 | 3,48E-01 | 9,40E-01 | 6,39E-01 | 3,97E-01 | 2,03E-01 | collagen, type XIV, alpha 1 |
| 12842 | *Col1a1* | 2,70E-01 | 4,58E-01 | 7,44E-01 | 3,78E-02 | 9,06E-03 | 9,00E-01 | 7,52E-01 | 3,35E-01 | 1,92E-01 | 4,88E-01 | collagen, type I, alpha 1 |
| 12843 | *Col1a2* | 3,04E-01 | 5,51E-01 | 1,92E-02 | 2,52E-02 | 1,51E-02 | 8,43E-01 | 8,77E-01 | 3,05E-01 | 1,61E-01 | 6,77E-01 | collagen, type I, alpha 2 |
| 12825 | *Col3a1* | 3,98E-02 | 6,68E-01 | 1,16E-02 | 2,91E-02 | 2,15E-02 | 5,54E-01 | 8,62E-01 | 4,65E-02 | 1,74E-01 | 1,87E-01 | collagen, type III, alpha 1 |
| 12832 | *Col5a2* | 1,23E-01 | 3,19E-01 | 1,46E-01 | 2,99E-02 | 1,10E-02 | 7,84E-01 | 6,50E-01 | 3,67E-02 | 1,61E-01 | 1,42E-01 | collagen, type V, alpha 2 |
| 12833 | *Col6a1* | 1,72E-01 | 7,50E-01 | 3,26E-02 | 9,70E-04 | 6,10E-02 | 6,14E-01 | 7,53E-01 | 3,00E-03 | 1,76E-01 | 2,18E-01 | collagen, type VI, alpha 1 |
| 12835 | *Col6a3* | 2,30E-01 | 2,42E-01 | 1,73E-02 | 4,69E-03 | 1,06E-02 | 4,75E-01 | 5,23E-01 | 8,75E-03 | 1,68E-01 | 2,10E-01 | collagen, type VI, alpha 3 |
| 12837 | *Col8a1* | 6,94E-01 | 8,44E-01 | 2,93E-01 | 7,25E-02 | 1,04E-02 | 7,27E-01 | 5,97E-01 | 1,64E-01 | 5,26E-01 | 9,50E-01 | collagen, type VIII, alpha 1 |
| *14268* | *Fn1* | 1,40E-01 | 2,42E-01 | 9,95E-01 | 7,33E-03 | 3,86E-02 | 7,44E-01 | 5,11E-01 | 9,40E-01 | 7,24E-01 | 4,19E-02 | fibronectin 1 |
| **Remodeling enzymes** | | | | | | |  | | | | |  |
| 16948 | *Lox* | 3,49E-02 | 6,62E-02 | 2,67E-01 | 2,34E-02 | 1,74E-02 | 2,24E-01 | 9,06E-02 | 3,55E-01 | 1,85E-01 | 5,32E-01 | lysyl oxidase |
| 268977 | *Ltbp1* | 5,00E-01 | 3,10E-01 | 5,73E-01 | 5,81E-03 | 7,00E-02 | 8,34E-01 | 9,29E-01 | 3,33E-01 | 7,81E-01 | 1,56E-01 | latent transforming growth factor beta binding protein 1 |
| 17387 | *Mmp14* | 2,53E-01 | 6,92E-02 | 5,85E-01 | 3,36E-03 | 5,55E-02 | 6,05E-01 | 1,85E-01 | 9,99E-01 | 1,87E-01 | 7,45E-02 | matrix metallopeptidase 14 (membrane-inserted) |
| 17390 | *Mmp2* | 4,64E-01 | 9,26E-01 | 2,01E-01 | 4,99E-02 | 2,19E-02 | 6,25E-01 | 6,31E-01 | 9,97E-01 | 4,17E-01 | 3,63E-01 | matrix metallopeptidase 2 |
| 17392 | *Mmp3* | 2,00E-01 | 8,24E-02 | 1,52E-01 | 5,79E-01 | 3,42E-01 | 5,67E-01 | 1,61E-01 | 2,01E-02 | 1,38E-01 | 4,49E-01 | matrix metallopeptidase 3 |
| 18791 | *Plat* | 3,31E-01 | 3,94E-01 | 1,10E-01 | 2,10E-03 | 1,46E-01 | 3,70E-01 | 4,12E-01 | 9,44E-01 | 7,52E-01 | 1,29E-01 | plasminogen activator, tissue |
| 18792 | *Plau* | 3,68E-01 | 1,26E-01 | 9,85E-01 | 1,27E-01 | 4,70E-01 | 4,13E-01 | 4,69E-02 | 7,86E-01 | 9,11E-02 | 7,96E-01 | plasminogen activator, urokinase |
| 18787 | *Serpine1* | 7,53E-04 | 3,30E-01 | 1,44E-01 | 4,18E-02 | 3,67E-01 | 3,31E-02 | 2,22E-01 | 3,74E-01 | 2,17E-01 | 7,79E-01 | serine (or cysteine) peptidase inhibitor, clade E, member 1 |
| 20720 | *Serpine2* | 2,79E-01 | 9,66E-01 | 7,88E-01 | 2,51E-02 | 3,47E-02 | 5,60E-01 | 6,54E-01 | 2,83E-02 | 3,48E-01 | 1,06E-01 | serine (or cysteine) peptidase inhibitor, clade E, member 2 |
| 21857 | *Timp1* | 2,38E-01 | 3,58E-01 | 4,16E-01 | 1,79E-02 | 7,95E-02 | 5,65E-02 | 3,70E-01 | 3,37E-02 | 2,59E-01 | 8,90E-02 | tissue inhibitor of metalloproteinase 1 |
| 21858 | *Timp2* | 4,49E-01 | 8,89E-01 | 4,38E-02 | 6,48E-03 | 4,66E-02 | 7,35E-01 | 6,98E-01 | 8,27E-01 | 6,72E-01 | 6,29E-01 | tissue inhibitor of metalloproteinase 2 |
| **Other regulatory factors and markers** | | | | | | |  | | | | |  |
| 12111 | *Bgn* | 6,37E-01 | 9,83E-01 | 4,13E-01 | 4,03E-04 | 1,26E-02 | 6,89E-01 | 8,45E-01 | 8,36E-01 | 2,48E-01 | 7,38E-01 | biglycan |
| 12153 | *Bmp1* | 4,22E-01 | 7,00E-01 | 9,24E-01 | 9,08E-04 | 6,21E-02 | 7,64E-01 | 9,99E-01 | 8,35E-01 | 4,30E-01 | 3,29E-01 | bone morphogenetic protein 1 |
| 18034 | *Nfkb2* | 1,74E-01 | 2,33E-01 | 3,43E-01 | 1,52E-02 | 1,70E-01 | 9,49E-02 | 2,92E-01 | 5,11E-01 | 3,07E-01 | 6,50E-01 | nuclear factor of kappa light polypeptide gene enhancer in B cells 2 |
| 18542 | *Pcolce* | 7,83E-01 | 5,71E-01 | 1,37E-01 | 2,26E-02 | 7,33E-02 | 1,07E-01 | 6,26E-01 | 1,53E-01 | 2,90E-01 | 7,39E-01 | procollagen C-endopeptidase enhancer protein |
| 21425 | *Tfeb* | 2,12E-01 | 8,64E-01 | 7,24E-03 | 1,84E-02 | 1,79E-02 | 9,78E-01 | 1,66E-01 | 5,95E-01 | 1,43E-01 | 4,02E-01 | transcription factor EB |
| 21803 | *Tgfb1* | 3,21E-01 | 2,71E-01 | 5,62E-02 | 3,70E-02 | 8,57E-02 | 2,05E-01 | 3,14E-01 | 1,34E-02 | 2,29E-01 | 2,20E-01 | transforming growth factor, beta 1 |
| 21813 | *Tgfbr2* | 3,41E-01 | 1,82E-01 | 2,39E-01 | 1,41E-03 | 1,96E-02 | 9,73E-01 | 6,12E-02 | 6,18E-01 | 4,73E-01 | 8,24E-01 | transforming growth factor, beta receptor II |
| 21838 | *Thy1* | 5,07E-01 | 3,69E-01 | 9,56E-01 | 1,13E-04 | 6,00E-02 | 8,43E-01 | 7,06E-01 | 9,42E-01 | 4,68E-01 | 9,04E-02 | thymus cell antigen 1, theta |
| 21897 | *Tlr1* | 7,11E-01 | 3,79E-01 | 6,50E-02 | 1,99E-02 | 1,84E-02 | 5,20E-01 | 2,98E-01 | 1,65E-01 | 1,92E-01 | 3,82E-01 | toll-like receptor 1 |
| 279572 | *Tlr13* | 6,19E-03 | 2,79E-01 | 2,48E-01 | 2,99E-02 | 2,15E-02 | 2,16E-01 | 3,54E-01 | 2,59E-02 | 2,17E-01 | 4,16E-01 | toll-like receptor 13 |
| 24088 | *Tlr2* | 6,73E-02 | 6,67E-01 | 6,83E-01 | 3,12E-02 | 1,63E-02 | 1,70E-01 | 1,62E-01 | 4,26E-01 | 2,76E-01 | 5,77E-01 | toll-like receptor 2 |
| 21898 | *Tlr4* | 1,25E-01 | 8,96E-02 | 6,73E-02 | 1,09E-02 | 2,17E-01 | 3,87E-01 | 3,89E-01 | 8,82E-01 | 2,23E-01 | 3,86E-01 | toll-like receptor 4 |
| 170743 | *Tlr7* | 2,84E-01 | 2,54E-01 | 9,80E-02 | 1,52E-02 | 6,55E-03 | 7,61E-01 | 8,47E-01 | 1,92E-01 | 2,03E-01 | 5,47E-01 | toll-like receptor 7 |
| 170744 | *Tlr8* | 8,57E-02 | 1,68E-02 | 1,58E-01 | 3,64E-03 | 1,01E-01 | 5,59E-01 | 3,84E-01 | 3,14E-01 | 1,93E-01 | 2,83E-01 | toll-like receptor 8 |
| 81897 | *Tlr9* | 3,45E-01 | 2,81E-01 | 5,74E-01 | 3,01E-02 | 7,90E-02 | 7,49E-02 | 1,84E-01 | 3,62E-02 | 1,69E-01 | 8,14E-01 | toll-like receptor 9 |
