# Supplementary material for: Diet-Induced Obesity Affects Muscle Regeneration After Murine Blunt Muscle Trauma—A Broad Spectrum Analysis
Source: Front Physiol. 2018 Jun 5;9:674. doi: 10.3389/fphys.2018.00674 (PMC5996306; doi:10.3389/fphys.2018.00674)
Supplement: Supplementary file 3 [file Table_3.docx]

Diet-induced obesity affects muscle regeneration after murine blunt muscle trauma – a broad spectrum analysis

Pengfei Xu^1†^, Jens-Uwe Werner^1†^, Sebastian Milerski^1^, Carmen Hamp^1^, Tatjana Kuzenko^1^, Markus Jähnert^2^, Pascal Gottmann^2^, Luisa de Roy^3^, Daniela Warnecke^3^, Alireza Abaei^4^, Annette Palmer^5^, Markus Huber-Lang^5^, Lutz Dürselen^3^, Volker Rasche^4^, Annette Schürmann^2^, Martin Wabitsch^6^*, Uwe Knippschild^1^*

* Correspondence: Prof. Dr. Uwe Knippschild, uwe.knippschild@uniklinik-ulm.de and Prof. Dr. Martin Wabitsch, martin.wabitsch@uniklinik-ulm.de

Sup. Tab. 3: P-values and descriptions for time lapse heatmap (hits ≥3) in lean and obese C57BL/6J mice. Red values are p ≤ 0.05.

|  |  | **Trauma versus Control** | | | | | | | | | |  |
| --- | --- | --- | --- | --- | --- | --- | --- | --- | --- | --- | --- | --- |
|  |  | **Normal** | | | | | **Obese** | | | | |  |
| **ID** | **Name** | **1h** | **6h** | **24h** | **3d** | **8d** | **1h** | **6h** | **24h** | **3d** | **8d** | **Description** |
| 107765 | *Ankrd1* | 2.96E-01 | 2.00E-01 | 3.36E-04 | 2.83E-03 | 1.96E-01 | 2.91E-02 | 4.55E-01 | 8.69E-02 | 7.84E-01 | 3.29E-01 | ankyrin repeat domain 1 (cardiac muscle) |
| 11820 | *App* | 3.44E-01 | 3.42E-01 | 3.51E-02 | 3.76E-03 | 1.26E-02 | 7.05E-01 | 4.77E-01 | 1.74E-01 | 3.21E-01 | 4.59E-01 | amyloid beta (A4) precursor protein |
| 11829 | *Aqp4* | 2.73E-01 | 6.33E-01 | 1.01E-01 | 2.74E-02 | 3.55E-02 | 6.78E-01 | 9.39E-01 | 1.98E-02 | 2.38E-01 | 2.42E-01 | aquaporin 4 |
| 12051 | *Bcl3* | 9.26E-03 | 1.69E-01 | 8.65E-02 | 4.23E-02 | 7.39E-02 | 3.80E-02 | 8.65E-02 | 2.09E-01 | 3.02E-01 | 3.30E-01 | B cell leukemia/lymphoma 3 |
| 12226 | *Btg1* | 9.20E-02 | 1.12E-01 | 2.98E-01 | 2.54E-03 | 4.14E-03 | 1.51E-02 | 1.78E-01 | 1.77E-01 | 1.43E-01 | 2.67E-02 | B cell translocation gene 1, anti-proliferative |
| 12267 | *C3ar1* | 1.86E-02 | 8.47E-02 | 2.01E-01 | 1.18E-02 | 4.11E-02 | 3.55E-01 | 1.44E-01 | 4.82E-02 | 2.17E-01 | 1.75E-01 | complement component 3a receptor 1 |
| 12363 | *Casp4* | 7.45E-03 | 1.93E-01 | 2.53E-02 | 2.42E-05 | 5.70E-02 | 1.88E-01 | 4.09E-02 | 8.53E-02 | 2.46E-01 | 1.83E-01 | caspase 4, apoptosis-related cysteine peptidase |
| 20305 | *Ccl6* | 1.51E-02 | 3.36E-02 | 8.83E-02 | 8.39E-03 | 2.15E-01 | 9.60E-01 | 1.04E-02 | 2.14E-01 | 2.69E-01 | 3.08E-01 | chemokine (C-C motif) ligand 6 |
| 20307 | *Ccl8* | 2.21E-01 | 2.70E-01 | 2.77E-02 | 3.71E-02 | 2.85E-02 | 4.54E-01 | 4.23E-01 | 1.37E-01 | 1.22E-01 | 4.66E-01 | chemokine (C-C motif) ligand 8 |
| 20308 | *Ccl9* | 2.93E-02 | 4.64E-02 | 1.49E-01 | 7.49E-03 | 1.54E-01 | 9.99E-01 | 4.37E-02 | 9.73E-02 | 2.55E-01 | 3.17E-01 | chemokine (C-C motif) ligand 9 |
| 23833 | *Cd52* | 1.19E-01 | 2.35E-01 | 8.22E-02 | 4.88E-03 | 1.62E-01 | 2.41E-02 | 3.92E-01 | 1.37E-02 | 2.15E-01 | 3.39E-01 | CD52 antigen |
| 12522 | *Cd83* | 2.52E-02 | 3.25E-01 | 6.28E-02 | 5.73E-03 | 5.57E-02 | 3.41E-02 | 3.33E-01 | 6.29E-01 | 2.71E-01 | 6.03E-01 | CD83 antigen |
| 14962 | *Cfb* | 5.48E-01 | 3.89E-01 | 5.36E-02 | 2.80E-02 | 4.30E-02 | 4.58E-01 | 5.95E-01 | 4.91E-02 | 1.49E-01 | 4.24E-01 | complement factor B |
| 76722 | *Ckmt2* | 8.87E-01 | 9.99E-01 | 1.93E-02 | 3.11E-02 | 1.91E-01 | 4.71E-01 | 9.39E-01 | 1.09E-01 | 4.33E-02 | 4.91E-01 | creatine kinase, mitochondrial 2 |
| 29876 | *Clic4* | 5.58E-02 | 3.63E-02 | 4.38E-01 | 2.34E-03 | 9.76E-03 | 2.22E-01 | 1.14E-01 | 3.84E-01 | 4.92E-01 | 1.49E-01 | chloride intracellular channel 4 (mitochondrial) |
| 12825 | *Col3a1* | 3.98E-02 | 6.68E-01 | 1.16E-02 | 2.91E-02 | 2.15E-02 | 5.54E-01 | 8.62E-01 | 4.65E-02 | 1.74E-01 | 1.87E-01 | collagen, type III, alpha 1 |
| 72042 | *Cotl1* | 9.70E-02 | 3.44E-01 | 1.46E-01 | 2.66E-03 | 4.38E-02 | 1.91E-01 | 1.92E-01 | 2.09E-02 | 2.96E-01 | 2.08E-01 | coactosin-like 1 (Dictyostelium) |
| 14219 | *Ctgf* | 9.33E-03 | 1.97E-01 | 1.04E-02 | 4.01E-02 | 2.57E-01 | 1.41E-01 | 2.35E-01 | 4.40E-01 | 2.88E-01 | 7.29E-01 | connective tissue growth factor |
| 13040 | *Ctss* | 1.96E-02 | 1.46E-01 | 1.66E-01 | 4.63E-04 | 6.79E-02 | 7.91E-01 | 3.30E-01 | 5.75E-03 | 1.82E-01 | 3.53E-01 | cathepsin S |
| 16007 | *Cyr61* | 6.96E-02 | 2.83E-01 | 1.99E-02 | 2.38E-02 | 3.54E-01 | 1.04E-02 | 1.03E-01 | 2.29E-01 | 6.84E-02 | 8.67E-01 | cysteine rich protein 61 |
| 72318 | *Cyth4* | 1.24E-01 | 3.84E-01 | 1.65E-02 | 2.47E-02 | 2.64E-02 | 6.02E-01 | 3.76E-01 | 2.25E-02 | 2.30E-01 | 2.39E-01 | cytohesin 4 |
| 13730 | *Emp1* | 5.81E-03 | 2.09E-02 | 1.87E-01 | 8.76E-04 | 1.09E-01 | 1.21E-01 | 1.68E-01 | 3.09E-01 | 2.91E-01 | 3.83E-01 | epithelial membrane protein 1 |
| 74155 | *Errfi1* | 2.23E-03 | 1.49E-01 | 1.89E-01 | 1.24E-02 | 1.89E-01 | 1.13E-02 | 3.12E-01 | 4.70E-01 | 1.23E-01 | 9.60E-01 | ERBB receptor feedback inhibitor 1 |
| 14130 | *Fcgr2b* | 4.91E-02 | 1.01E-02 | 3.53E-02 | 2.46E-03 | 7.28E-02 | 5.89E-01 | 2.30E-01 | 1.78E-01 | 1.52E-01 | 2.60E-01 | Fc receptor, IgG, low affinity IIb |
| 14190 | *Fgl2* | 2.41E-02 | 4.49E-02 | 6.25E-01 | 6.12E-02 | 7.14E-01 | 4.60E-02 | 1.55E-01 | 5.49E-01 | 2.55E-01 | 2.80E-01 | fibrinogen-like protein 2 |
| 14268 | *Fn1* | 1.40E-01 | 2.42E-01 | 9.95E-01 | 7.33E-03 | 3.86E-02 | 7.44E-01 | 5.11E-01 | 9.40E-01 | 7.24E-01 | 4.19E-02 | fibronectin 1 |
| 14545 | *Gdap1* | 5.24E-01 | 2.21E-01 | 3.17E-02 | 7.08E-03 | 7.21E-03 | 8.74E-01 | 6.48E-01 | 8.43E-01 | 2.66E-01 | 4.29E-01 | ganglioside-induced differentiation-associated-protein 1 |
| 14955 | *H19* | 7.39E-01 | 7.72E-01 | 2.07E-02 | 3.46E-02 | 3.02E-03 | 6.01E-01 | 7.11E-01 | 1.43E-01 | 1.57E-01 | 3.92E-01 | H19, imprinted maternally expressed transcript |
| 15430 | *Hoxd10* | 4.40E-01 | 9.29E-01 | 1.76E-02 | 1.77E-01 | 3.79E-01 | 4.87E-01 | 8.05E-01 | 4.67E-02 | 4.16E-02 | 3.71E-01 | homeobox D10 |
| 319415 | *Hs3st5* | 2.09E-01 | 2.39E-01 | 8.64E-01 | 3.66E-02 | 8.83E-03 | 3.68E-01 | 1.82E-02 | 1.77E-01 | 6.45E-01 | 1.17E-01 | heparan sulfate (glucosamine) 3-O-sulfotransferase 5 |
| 15894 | *Icam1* | 9.75E-02 | 3.51E-01 | 7.63E-01 | 3.15E-03 | 3.23E-02 | 2.33E-02 | 1.04E-01 | 8.41E-02 | 2.25E-01 | 3.82E-01 | intercellular adhesion molecule 1 |
| 15959 | *Ifit3* | 7.60E-02 | 4.28E-01 | 2.73E-01 | 9.42E-04 | 4.01E-01 | 1.28E-02 | 5.85E-01 | 3.26E-02 | 3.02E-01 | 3.15E-01 | interferon-induced protein with tetratricopeptide repeats 3 |
| 80719 | *Igsf6* | 4.71E-03 | 2.48E-01 | 8.48E-02 | 2.46E-02 | 1.57E-01 | 2.02E-02 | 4.30E-01 | 1.97E-01 | 2.18E-01 | 2.97E-01 | immunoglobulin superfamily, member 6 |
| 73914 | *Irak3* | 2.73E-02 | 8.56E-03 | 4.86E-01 | 6.41E-04 | 8.05E-02 | 2.59E-01 | 2.39E-01 | 6.40E-01 | 5.53E-01 | 2.02E-01 | interleukin-1 receptor-associated kinase 3 |
| 16402 | *Itga5* | 2.58E-02 | 2.68E-01 | 4.54E-01 | 1.52E-04 | 1.22E-01 | 4.53E-02 | 3.74E-01 | 6.43E-01 | 4.23E-01 | 6.13E-01 | integrin alpha 5 (fibronectin receptor alpha) |
| 16414 | *Itgb2* | 1.31E-02 | 1.63E-01 | 1.77E-01 | 3.56E-02 | 1.71E-01 | 2.32E-02 | 3.26E-01 | 2.92E-02 | 2.26E-01 | 3.41E-01 | integrin beta 2 |
| 277396 | *Klhl23* | 2.59E-01 | 3.10E-01 | 2.32E-02 | 1.49E-02 | 3.87E-01 | 8.96E-01 | 3.38E-01 | 2.29E-01 | 4.44E-02 | 5.05E-01 | kelch-like 23 |
| 244864 | *Layn* | 8.73E-01 | 1.43E-02 | 4.41E-01 | 3.68E-04 | 6.08E-03 | 9.33E-01 | 2.04E-01 | 1.08E-01 | 1.12E-01 | 3.49E-01 | layilin |
| 18826 | *Lcp1* | 7.98E-03 | 2.02E-01 | 1.61E-01 | 1.65E-02 | 1.21E-01 | 9.26E-03 | 3.09E-01 | 4.58E-02 | 2.18E-01 | 2.52E-01 | lymphocyte cytosolic protein 1 |
| 16822 | *Lcp2* | 2.52E-02 | 1.76E-01 | 3.98E-02 | 2.87E-02 | 8.26E-02 | 4.36E-02 | 3.13E-01 | 9.92E-02 | 3.05E-01 | 3.31E-01 | lymphocyte cytosolic protein 2 |
| 16854 | *Lgals3* | 1.15E-01 | 1.22E-01 | 3.45E-02 | 1.63E-02 | 1.39E-02 | 7.79E-01 | 1.26E-01 | 1.01E-02 | 1.88E-01 | 2.42E-01 | lectin, galactose binding, soluble 3 |
| 56722 | *Litaf* | 4.75E-02 | 6.34E-02 | 5.02E-01 | 2.77E-03 | 9.64E-02 | 4.19E-02 | 7.77E-02 | 7.94E-02 | 2.45E-01 | 3.61E-01 | LPS-induced TN factor |
| 16948 | *Lox* | 3.49E-02 | 6.62E-02 | 2.67E-01 | 2.34E-02 | 1.74E-02 | 2.24E-01 | 9.06E-02 | 3.55E-01 | 1.85E-01 | 5.32E-01 | lysyl oxidase |
| 16979 | *Lrrn1* | 2.38E-01 | 7.93E-02 | 1.21E-02 | 4.27E-03 | 2.13E-02 | 2.81E-01 | 7.04E-01 | 8.50E-01 | 1.03E-01 | 9.13E-01 | leucine rich repeat protein 1, neuronal |
| 26410 | *Map3k8* | 2.43E-02 | 2.98E-01 | 3.05E-01 | 3.12E-02 | 2.32E-02 | 9.82E-02 | 2.83E-01 | 5.04E-01 | 3.81E-01 | 9.14E-01 | mitogen-activated protein kinase kinase kinase 8 |
| 59090 | *Midn* | 3.35E-02 | 1.23E-01 | 8.51E-01 | 9.37E-03 | 2.17E-02 | 7.23E-02 | 4.60E-01 | 2.97E-01 | 7.76E-01 | 6.14E-01 | midnolin |
| 17476 | *Mpeg1* | 1.15E-01 | 2.63E-01 | 1.84E-01 | 2.00E-02 | 4.46E-02 | 2.64E-01 | 4.03E-01 | 2.79E-03 | 1.80E-01 | 3.58E-01 | macrophage expressed gene 1 |
| 73656 | *Ms4a6c* | 3.78E-02 | 9.35E-02 | 1.43E-01 | 9.78E-03 | 9.13E-02 | 6.74E-01 | 2.94E-01 | 3.30E-02 | 1.92E-01 | 3.73E-01 | membrane-spanning 4-domains, subfamily A, member 6C |
| 74843 | *Mss51* | 3.87E-01 | 3.77E-01 | 1.36E-02 | 1.50E-02 | 4.26E-03 | 5.75E-01 | 9.07E-01 | 9.82E-01 | 8.87E-01 | 1.75E-01 | MSS51 mitochondrial translational activator |
| 17748 | *Mt1* | 6.89E-02 | 1.11E-02 | 1.58E-01 | 1.45E-02 | 8.86E-03 | 4.36E-02 | 2.06E-01 | 9.55E-02 | 3.40E-01 | 7.70E-01 | metallothionein 1 |
| 18198 | *Musk* | 4.20E-01 | 7.34E-01 | 1.24E-01 | 9.59E-03 | 3.12E-01 | 7.08E-01 | 4.41E-01 | 3.31E-02 | 2.16E-03 | 2.05E-01 | muscle, skeletal, receptor tyrosine kinase |
| 17874 | *Myd88* | 2.93E-02 | 9.94E-02 | 2.00E-01 | 1.79E-02 | 2.74E-03 | 4.56E-02 | 2.71E-01 | 5.60E-01 | 4.22E-01 | 1.16E-01 | myeloid differentiation primary response gene 88 |
| 228785 | *Mylk2* | 1.26E-01 | 4.33E-01 | 1.47E-03 | 6.49E-04 | 2.40E-02 | 7.77E-01 | 6.93E-01 | 1.29E-01 | 9.58E-01 | 1.93E-01 | myosin, light polypeptide kinase 2, skeletal muscle |
| 17937 | *Nab2* | 4.94E-01 | 3.18E-01 | 2.98E-01 | 9.75E-03 | 5.67E-03 | 6.03E-03 | 4.31E-01 | 8.23E-02 | 7.38E-01 | 3.63E-01 | Ngfi-A binding protein 2 |
| 105855 | *Nckap1l* | 2.82E-02 | 1.92E-01 | 8.46E-02 | 1.48E-02 | 7.43E-02 | 3.73E-01 | 1.77E-01 | 4.19E-02 | 2.32E-01 | 4.50E-01 | NCK associated protein 1 like |
| 18414 | *Osmr* | 2.24E-02 | 3.17E-02 | 5.87E-01 | 1.29E-02 | 9.91E-02 | 2.64E-01 | 1.30E-01 | 3.12E-01 | 4.45E-01 | 7.83E-01 | oncostatin M receptor |
| 67041 | *Oxct1* | 8.26E-01 | 4.55E-01 | 8.99E-04 | 1.40E-02 | 9.31E-02 | 5.25E-01 | 8.87E-01 | 4.64E-02 | 1.40E-01 | 5.42E-01 | 3-oxoacid CoA transferase 1 |
| 170768 | *Pfkfb3* | 3.06E-02 | 6.11E-01 | 7.23E-01 | 3.01E-02 | 1.10E-01 | 7.69E-01 | 2.72E-01 | 4.70E-01 | 2.58E-02 | 6.95E-01 | 6-phosphofructo-2-kinase/fructose-2,6-biphosphatase 3 |
| 66681 | *Pgm1* | 3.74E-02 | 1.47E-01 | 6.03E-01 | 6.33E-03 | 7.90E-02 | 3.60E-02 | 2.38E-01 | 4.54E-01 | 6.11E-01 | 1.26E-01 | phosphoglucomutase 1 |
| 83490 | *Pik3ap1* | 8.97E-02 | 5.08E-01 | 2.78E-01 | 2.45E-02 | 2.77E-02 | 7.13E-01 | 1.13E-01 | 3.47E-03 | 1.83E-01 | 4.57E-01 | phosphoinositide-3-kinase adaptor protein 1 |
| 234779 | *Plcg2* | 1.19E-01 | 5.77E-01 | 3.83E-01 | 4.02E-02 | 3.59E-02 | 1.71E-01 | 5.27E-01 | 6.51E-03 | 3.25E-02 | 4.69E-02 | phospholipase C, gamma 2 |
| 67448 | *Plxdc2* | 2.44E-01 | 6.14E-01 | 3.85E-02 | 4.01E-03 | 1.93E-02 | 5.75E-01 | 5.87E-01 | 1.35E-01 | 5.06E-01 | 3.05E-01 | plexin domain containing 2 |
| 243382 | *Ppm1k* | 5.16E-01 | 2.38E-01 | 2.52E-02 | 1.52E-02 | 3.25E-02 | 9.18E-01 | 2.09E-01 | 5.02E-02 | 1.39E-01 | 6.97E-01 | protein phosphatase 1K (PP2C domain containing) |
| 19264 | *Ptprc* | 3.82E-02 | 2.38E-01 | 1.16E-01 | 1.45E-03 | 4.14E-02 | 1.37E-02 | 3.53E-01 | 7.63E-02 | 2.20E-01 | 3.45E-01 | protein tyrosine phosphatase, receptor type, C |
| 67442 | *Retsat* | 6.28E-01 | 3.64E-01 | 1.46E-02 | 3.83E-02 | 1.90E-01 | 6.96E-01 | 6.75E-01 | 1.98E-02 | 2.66E-02 | 7.61E-01 | retinol saturase (all trans retinol 13,14 reductase) |
| 74194 | *Rnd3* | 2.36E-02 | 2.23E-01 | 3.25E-01 | 8.39E-03 | 8.67E-03 | 1.20E-01 | 8.14E-02 | 7.50E-01 | 5.81E-01 | 6.63E-01 | Rho family GTPase 3 |
| 672511 | *Rnf213* | 6.49E-01 | 3.01E-01 | 6.70E-02 | 9.60E-03 | 4.13E-02 | 6.61E-01 | 2.76E-01 | 2.93E-02 | 2.03E-01 | 2.50E-01 | ring finger protein 213 |
| 20194 | *S100a10* | 2.36E-02 | 4.13E-02 | 8.87E-01 | 1.03E-03 | 2.22E-01 | 1.57E-01 | 2.08E-01 | 8.02E-02 | 2.77E-01 | 1.77E-01 | S100 calcium binding protein A10 (calpactin) |
| 20970 | *Sdc3* | 7.89E-01 | 3.86E-01 | 6.09E-02 | 1.50E-02 | 3.69E-02 | 2.51E-01 | 8.97E-01 | 3.55E-02 | 2.56E-01 | 2.11E-01 | syndecan 3 |
| 20345 | *Selplg* | 3.47E-02 | 3.17E-01 | 2.59E-01 | 1.23E-02 | 1.00E-01 | 2.73E-02 | 3.70E-01 | 1.10E-01 | 1.76E-01 | 2.80E-01 | selectin, platelet (p-selectin) ligand |
| 18787 | *Serpine1* | 7.53E-04 | 3.30E-01 | 1.44E-01 | 4.18E-02 | 3.67E-01 | 3.31E-02 | 2.22E-01 | 3.74E-01 | 2.17E-01 | 7.79E-01 | serine (or cysteine) peptidase inhibitor, clade E, member 1 |
| 100217426 | *Snord49b* | 9.23E-01 | 8.58E-01 | 9.43E-02 | 1.11E-02 | 4.34E-02 | 9.78E-01 | 3.13E-01 | 8.38E-03 | 2.54E-01 | 6.78E-01 | small nucleolar RNA, C/D box 49B |
| 234214 | *Sorbs2* | 7.97E-01 | 7.46E-01 | 5.61E-01 | 4.32E-02 | 1.50E-03 | 9.42E-01 | 6.81E-01 | 6.81E-01 | 1.99E-02 | 6.78E-01 | sorbin and SH3 domain containing 2 |
| 98267 | *Stk17b* | 9.44E-02 | 3.28E-01 | 1.79E-01 | 1.95E-02 | 4.97E-02 | 2.65E-02 | 7.31E-01 | 6.17E-01 | 7.20E-02 | 2.83E-01 | serine/threonine kinase 17b (apoptosis-inducing) |
| 21825 | *Thbs1* | 1.12E-02 | 2.17E-01 | 1.91E-02 | 1.83E-02 | 3.46E-01 | 6.91E-02 | 6.42E-02 | 8.10E-02 | 2.31E-01 | 7.26E-01 | thrombospondin 1 |
| 279572 | *Tlr13* | 6.19E-03 | 2.79E-01 | 2.48E-01 | 2.99E-02 | 2.15E-02 | 2.16E-01 | 3.54E-01 | 2.59E-02 | 2.17E-01 | 4.16E-01 | toll-like receptor 13 |
| 21923 | *Tnc* | 1.25E-01 | 2.16E-01 | 1.79E-02 | 3.95E-02 | 2.84E-02 | 2.70E-01 | 2.77E-01 | 7.82E-01 | 3.61E-01 | 9.81E-01 | tenascin C |
| 21933 | *Tnfrsf10b* | 1.02E-01 | 8.93E-02 | 1.34E-01 | 3.23E-02 | 2.96E-01 | 2.25E-02 | 7.18E-03 | 5.76E-01 | 3.70E-01 | 8.08E-01 | tumor necrosis factor receptor superfamily, member 10b |
| 69480 | *Ttc9* | 8.52E-01 | 3.47E-01 | 1.73E-02 | 5.04E-03 | 3.37E-02 | 4.61E-01 | 5.03E-01 | 4.25E-01 | 1.22E-01 | 4.94E-01 | tetratricopeptide repeat domain 9 |
| 22235 | *Ugdh* | 8.03E-03 | 3.31E-02 | 6.15E-01 | 3.66E-03 | 8.08E-02 | 7.32E-02 | 1.85E-01 | 8.90E-01 | 4.75E-01 | 2.88E-01 | UDP-glucose dehydrogenase |
| 381066 | *Zfp948* | 2.15E-02 | 2.64E-01 | 1.55E-01 | 8.61E-04 | 5.90E-02 | 3.41E-02 | 3.30E-01 | 7.47E-01 | 4.71E-02 | 3.87E-01 | zinc finger protein 948 |
