# Supplementary material for: Diet-Induced Obesity Affects Muscle Regeneration After Murine Blunt Muscle Trauma—A Broad Spectrum Analysis
Source: Front Physiol. 2018 Jun 5;9:674. doi: 10.3389/fphys.2018.00674 (PMC5996306; doi:10.3389/fphys.2018.00674)
Supplement: Supplementary file 4 [file Table_4.docx]

Diet-induced obesity affects muscle regeneration after murine blunt muscle trauma – a broad spectrum analysis

Pengfei Xu^1†^, Jens-Uwe Werner^1†^, Sebastian Milerski^1^, Carmen Hamp^1^, Tatjana Kuzenko^1^, Markus Jähnert^2^, Pascal Gottmann^2^, Luisa de Roy^3^, Daniela Warnecke^3^, Alireza Abaei^4^, Annette Palmer^5^, Markus Huber-Lang^5^, Lutz Dürselen^3^, Volker Rasche^4^, Annette Schürmann^2^, Martin Wabitsch^6^*, Uwe Knippschild^1^*

* Correspondence: Prof. Dr. Uwe Knippschild, uwe.knippschild@uniklinik-ulm.de and Prof. Dr. Martin Wabitsch, martin.wabitsch@uniklinik-ulm.de

Sup. Tab. 4: P-values and descriptions for complement-, Wnt- and satellite cell-related genes in lean and obese C57BL/6J mice. Red values are p ≤ 0.05.

|  |  | **Trauma versus Control** | | | | | | | | | |  |
| --- | --- | --- | --- | --- | --- | --- | --- | --- | --- | --- | --- | --- |
|  |  | **Normal** | | | | | **Obese** | | | | |  |
| **ID** | **Name** | **1h** | **6h** | **24h** | **3d** | **8d** | **1h** | **6h** | **24h** | **3d** | **8d** | **Description** |
| **Complement** | |  |  |  |  |  |  |  |  |  |  |  |
| 12259 | *C1qa* | 3.64E-01 | 6.21E-01 | 3.80E-02 | 7.50E-03 | 9.45E-02 | 2.02E-01 | 6.14E-01 | 3.90E-01 | 2.07E-01 | 4.14E-01 | c. component 1, q subcomponent, alpha polypeptide |
| 12260 | *C1qb* | 3.18E-01 | 3.56E-01 | 8.60E-03 | 6.32E-03 | 1.20E-01 | 7.95E-01 | 9.94E-01 | 2.81E-01 | 2.43E-01 | 2.72E-01 | c. component 1, q subcomponent, beta polypeptide |
| 12262 | *C1qc* | 5.84E-01 | 6.53E-01 | 2.57E-02 | 6.99E-03 | 4.22E-02 | 4.55E-01 | 6.33E-01 | 3.99E-01 | 2.29E-01 | 3.68E-01 | c. component 1, q subcomponent, C chain |
| 81799 | *C1qtnf3* | 5.02E-01 | 5.71E-01 | 5.82E-01 | 4.27E-02 | 1.39E-01 | 6.50E-01 | 7.72E-01 | 5.08E-01 | 4.38E-01 | 3.29E-01 | C1q and tumor necrosis factor related protein 3 |
| 12267 | *C3ar1* | 1.86E-02 | 8.47E-02 | 2.01E-01 | 1.18E-02 | 4.11E-02 | 3.55E-01 | 1.44E-01 | 4.82E-02 | 2.17E-01 | 1.75E-01 | c. component 3a receptor 1 |
| 12273 | *C5ar1* | 6.71E-02 | 1.46E-01 | 2.37E-01 | 1.38E-02 | 9.83E-02 | 9.44E-03 | 2.14E-01 | 4.14E-03 | 2.14E-01 | 7.50E-01 | c. component 5a receptor 1 |
| 14962 | *Cfb* | 5.48E-01 | 3.89E-01 | 5.36E-02 | 2.80E-02 | 4.30E-02 | 4.58E-01 | 5.95E-01 | 4.91E-02 | 1.49E-01 | 4.24E-01 | c. factor B |
| 74145 | *F13a1* | 2.14E-01 | 2.98E-01 | 6.54E-02 | 2.73E-02 | 1.00E-01 | 6.87E-01 | 1.79E-01 | 3.82E-01 | 1.79E-01 | 6.31E-01 | coagulation factor XIII, A1 subunit |
| **Wnt** | |  |  |  |  |  |  |  |  |  |  |  |
| 12505 | *Cd44* | 5.95E-02 | 2.63E-01 | 1.84E-01 | 5.20E-03 | 9.78E-02 | 6.74E-02 | 2.93E-01 | 1.40E-01 | 3.67E-01 | 4.66E-02 | CD44 antigen |
| 12995 | *Csnk2a1* | 5.85E-01 | 9.78E-01 | 2.07E-02 | 5.30E-03 | 2.92E-02 | 6.42E-01 | 4.27E-01 | 9.18E-02 | 8.37E-01 | 5.20E-01 | casein kinase 2, alpha 1 polypeptide |
| 12385 | *Ctnna1* | 2.14E-01 | 3.95E-01 | 1.66E-01 | 9.11E-03 | 3.94E-02 | 8.90E-01 | 2.08E-01 | 2.80E-01 | 1.19E-01 | 6.53E-01 | catenin (cadherin associated protein), alpha 1 |
| 216033 | *Ctnna3* | 8.43E-01 | 8.69E-01 | 1.40E-01 | 4.19E-02 | 4.89E-02 | 7.13E-01 | 9.06E-01 | 9.15E-01 | 1.20E-01 | 6.14E-01 | catenin (cadherin associated protein), alpha 3 |
| 67087 | *Ctnnbip1* | 3.00E-01 | 1.36E-01 | 1.74E-01 | 5.94E-03 | 1.69E-01 | 1.90E-01 | 6.06E-01 | 4.12E-01 | 6.87E-01 | 2.42E-01 | catenin beta interacting protein 1 |
| 54601 | *Foxo4* | 7.59E-01 | 3.78E-01 | 1.09E-01 | 1.99E-02 | 2.39E-03 | 2.19E-01 | 4.65E-01 | 3.93E-01 | 9.33E-01 | 8.26E-01 | forkhead box O4 |
| 26409 | *Map3k7* | 6.66E-01 | 6.37E-01 | 3.04E-01 | 7.81E-04 | 4.69E-02 | 2.50E-01 | 7.37E-01 | 2.29E-01 | 9.89E-01 | 9.06E-01 | mitogen-activated protein kinase kinase kinase 7 |
| 26410 | *Map3k8* | 2.43E-02 | 2.98E-01 | 3.05E-01 | 3.12E-02 | 2.32E-02 | 9.82E-02 | 2.83E-01 | 5.04E-01 | 3.81E-01 | 9.14E-01 | mitogen-activated protein kinase kinase kinase 8 |
| 17169 | *Mark3* | 2.01E-01 | 3.08E-01 | 2.51E-01 | 9.00E-03 | 6.62E-03 | 9.92E-01 | 2.37E-01 | 4.34E-01 | 6.48E-01 | 9.86E-01 | MAP/microtubule affinity regulating kinase 3 |
| 17869 | *Myc* | 1.48E-02 | 1.94E-01 | 6.68E-02 | 5.38E-02 | 3.38E-01 | 4.57E-02 | 2.61E-01 | 1.36E-01 | 9.08E-01 | 1.88E-01 | myelocytomatosis oncogene |
| 20377 | *Sfrp1* | 9.46E-01 | 7.49E-01 | 2.41E-01 | 3.21E-02 | 3.46E-02 | 5.36E-01 | 8.02E-01 | 3.75E-01 | 1.30E-01 | 8.53E-01 | secreted frizzled-related protein 1 |
| 20319 | *Sfrp2* | 9.40E-01 | 8.80E-01 | 6.79E-01 | 3.14E-02 | 2.29E-02 | 6.53E-01 | 7.30E-01 | 4.73E-01 | 2.95E-01 | 3.71E-01 | secreted frizzled-related protein 2 |
| 20583 | *Snai2* | 9.49E-01 | 7.65E-01 | 8.90E-01 | 5.73E-03 | 1.99E-01 | 4.58E-01 | 8.35E-01 | 6.20E-01 | 7.50E-01 | 6.09E-01 | snail family zinc finger 2 |
| 21406 | *Tcf12* | 6.95E-01 | 4.66E-01 | 3.09E-01 | 6.54E-03 | 7.24E-03 | 4.09E-01 | 4.84E-01 | 7.10E-01 | 4.67E-01 | 9.02E-01 | transcription factor 12 |
| 21423 | *Tcf3* | 9.30E-01 | 3.78E-01 | 1.27E-01 | 6.46E-03 | 1.45E-02 | 4.70E-01 | 6.93E-01 | 3.36E-01 | 7.28E-01 | 7.63E-01 | transcription factor 3 |
| 21803 | *Tgfb1* | 3.21E-01 | 2.71E-01 | 5.62E-02 | 3.70E-02 | 8.57E-02 | 2.05E-01 | 3.14E-01 | 1.34E-02 | 2.29E-01 | 2.20E-01 | transforming growth factor, beta 1 |
| 21804 | *Tgfb1i1* | 3.23E-01 | 9.96E-01 | 8.66E-02 | 4.57E-02 | 1.29E-01 | 2.11E-01 | 3.30E-01 | 2.20E-01 | 6.86E-01 | 6.83E-01 | transforming growth factor beta 1 induced transcript 1 |
| 21810 | *Tgfbi* | 3.77E-01 | 8.39E-02 | 5.58E-01 | 2.24E-03 | 4.06E-02 | 3.66E-01 | 5.54E-01 | 1.36E-01 | 3.09E-01 | 6.78E-01 | transforming growth factor, beta induced |
| 21812 | *Tgfbr1* | 7.35E-01 | 3.50E-01 | 1.68E-01 | 1.41E-02 | 5.35E-01 | 4.24E-01 | 5.04E-01 | 6.95E-01 | 9.99E-01 | 2.66E-01 | transforming growth factor, beta receptor I |
| 21813 | *Tgfbr2* | 3.41E-01 | 1.82E-01 | 2.39E-01 | 1.41E-03 | 1.96E-02 | 9.73E-01 | 6.12E-02 | 6.18E-01 | 4.73E-01 | 8.24E-01 | transforming growth factor, beta receptor II |
| **Satellite cells** | |  |  |  |  |  |  |  |  |  |  |  |
| 12490 | *Cd34* | 3.53E-01 | 7.46E-01 | 2.71E-01 | 4.12E-03 | 1.72E-01 | 8.59E-01 | 6.52E-01 | 7.69E-01 | 7.81E-01 | 5.29E-01 | CD34 antigen |
| 12552 | *Cdh11* | 3.07E-01 | 5.27E-01 | 1.58E-01 | 5.44E-03 | 1.78E-02 | 6.82E-01 | 8.65E-01 | 1.34E-01 | 4.41E-01 | 4.71E-01 | cadherin 11 |
| 12554 | *Cdh13* | 1.94E-01 | 5.78E-01 | 3.62E-01 | 5.77E-01 | 3.05E-02 | 6.75E-01 | 4.39E-01 | 4.66E-02 | 3.15E-01 | 7.92E-01 | cadherin 13 |
| 12555 | *Cdh15* | 3.53E-01 | 7.19E-01 | 9.57E-01 | 9.33E-03 | 4.90E-01 | 7.97E-01 | 4.09E-01 | 9.83E-01 | 6.29E-01 | 2.79E-01 | cadherin 15 |
| 12558 | *Cdh2* | 8.29E-01 | 7.14E-01 | 9.66E-01 | 3.30E-02 | 6.37E-02 | 9.65E-01 | 3.49E-01 | 5.88E-01 | 4.80E-01 | 2.51E-01 | cadherin 2 |
| 12767 | *Cxcr4* | 2.93E-01 | 5.73E-01 | 2.92E-01 | 1.55E-02 | 1.41E-01 | 8.12E-02 | 7.14E-01 | 1.81E-01 | 3.77E-01 | 4.55E-01 | chemokine (C-X-C motif) receptor 4 |
| 16399 | *Itga2b* | 9.44E-02 | 4.72E-01 | 4.41E-01 | 6.43E-02 | 2.08E-02 | 5.13E-01 | 5.30E-01 | 2.81E-01 | 2.77E-01 | 4.13E-01 | integrin alpha 2b |
| 16402 | *Itga5* | 2.58E-02 | 2.68E-01 | 4.54E-01 | 1.52E-04 | 1.22E-01 | 4.53E-02 | 3.74E-01 | 6.43E-01 | 4.23E-01 | 6.13E-01 | integrin alpha 5 (fibronectin receptor alpha) |
| 16404 | *Itga7* | 9.46E-01 | 4.06E-01 | 3.63E-01 | 1.67E-03 | 2.64E-02 | 5.98E-01 | 7.29E-01 | 9.11E-01 | 2.86E-02 | 7.70E-01 | integrin alpha 7 |
| 16408 | *Itgal* | 1.14E-02 | 3.76E-01 | 5.37E-01 | 3.78E-03 | 2.71E-02 | 2.10E-02 | 3.73E-01 | 3.03E-01 | 3.32E-01 | 6.48E-01 | integrin alpha L |
| 16409 | *Itgam* | 3.22E-02 | 1.15E-01 | 6.33E-02 | 2.25E-02 | 1.93E-01 | 1.15E-01 | 3.14E-01 | 1.66E-01 | 2.41E-01 | 2.99E-01 | integrin alpha M |
| 16410 | *Itgav* | 2.59E-01 | 6.22E-01 | 3.06E-01 | 5.43E-03 | 3.41E-02 | 7.63E-01 | 4.31E-01 | 2.11E-01 | 3.14E-01 | 3.07E-02 | integrin alpha V |
| 16411 | *Itgax* | 2.21E-01 | 8.17E-01 | 6.46E-01 | 7.21E-02 | 1.87E-02 | 1.49E-01 | 8.70E-01 | 2.68E-01 | 9.89E-01 | 1.48E-01 | integrin alpha X |
| 16412 | *Itgb1* | 1.09E-01 | 1.66E-01 | 6.69E-03 | 2.56E-04 | 2.69E-02 | 2.30E-01 | 2.81E-01 | 6.39E-02 | 3.46E-01 | 1.03E-01 | integrin beta 1 (fibronectin receptor beta) |
| 16414 | *Itgb2* | 1.31E-02 | 1.63E-01 | 1.77E-01 | 3.56E-02 | 1.71E-01 | 2.32E-02 | 3.26E-01 | 2.92E-02 | 2.26E-01 | 3.41E-01 | integrin beta 2 |
| 16419 | *Itgb5* | 3.45E-01 | 2.46E-01 | 7.75E-03 | 1.28E-02 | 3.47E-01 | 3.31E-01 | 7.96E-01 | 7.56E-02 | 1.94E-01 | 9.27E-01 | integrin beta 5 |
| 17295 | *Met* | 3.50E-01 | 1.68E-01 | 9.09E-01 | 2.38E-02 | 3.53E-01 | 7.11E-01 | 4.61E-01 | 2.76E-01 | 1.90E-01 | 6.72E-01 | met proto-oncogene |
| 17877 | *Myf5* | 8.11E-01 | 7.80E-01 | 3.00E-01 | 6.74E-03 | 3.46E-02 | 3.07E-01 | 3.00E-01 | 2.55E-01 | 3.67E-01 | 3.46E-01 | myogenic factor 5 |
| 17878 | *Myf6* | 5.67E-01 | 3.83E-03 | 9.08E-01 | 9.72E-02 | 2.56E-01 | 7.31E-02 | 2.11E-01 | 5.66E-01 | 9.85E-01 | 3.76E-01 | myogenic factor 6 |
| 228785 | *Mylk2* | 1.26E-01 | 4.33E-01 | 1.47E-03 | 6.49E-04 | 2.40E-02 | 7.77E-01 | 6.93E-01 | 1.29E-01 | 9.58E-01 | 1.93E-01 | myosin, light polypeptide kinase 2, skeletal muscle |
| 17927 | *Myod1* | 9.07E-01 | 3.91E-01 | 4.80E-01 | 2.86E-02 | 3.68E-02 | 5.67E-01 | 3.95E-01 | 6.83E-01 | 3.94E-01 | 2.74E-01 | myogenic differentiation 1 |
| 17928 | *Myog* | 5.96E-01 | 4.49E-01 | 4.50E-02 | 1.60E-03 | 1.22E-01 | 4.60E-01 | 4.43E-01 | 1.98E-02 | 3.91E-03 | 3.10E-01 | myogenin |
