# Supplementary material for: Diet-Induced Obesity Affects Muscle Regeneration After Murine Blunt Muscle Trauma—A Broad Spectrum Analysis
Source: Front Physiol. 2018 Jun 5;9:674. doi: 10.3389/fphys.2018.00674 (PMC5996306; doi:10.3389/fphys.2018.00674)
Supplement: Supplementary file 5 [file Image_1.pdf]

Diet-induced obesity affects muscle regeneration after murine blunt muscle trauma – a broad spectrum analysis

Pengfei Xu[1†], Jens-Uwe Werner[1†], Sebastian Milerski[1], Carmen Hamp[1], Tatjana Kuzenko[1], Markus Jähnert[2], Pascal Gottmann[2], Luisa de Roy[3], Daniela Warnecke[3], Alireza Abaei[4], Annette Palmer[5], Markus Huber-Lang[5], Lutz Dürselen[3], Volker Rasche[4], Annette Schürmann[2], Martin Wabitsch[6]*, Uwe Knippschild[1]*

* Correspondence: Prof. Dr. Uwe Knippschild, uwe.knippschild@uniklinik-ulm.de and Prof. Dr. Martin Wabitsch, martin.wabitsch@uniklinik-ulm.de

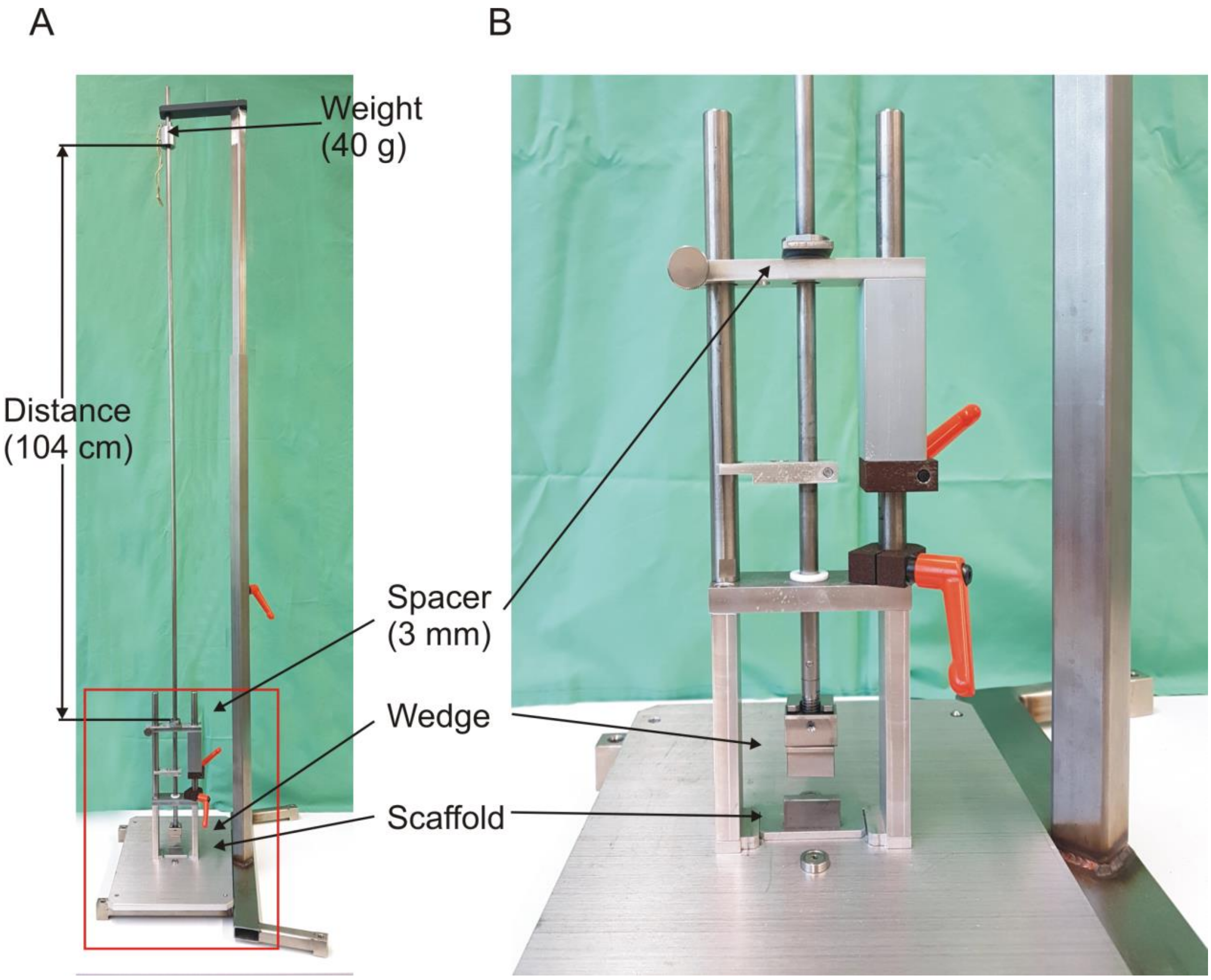

Sup. Fig. 1: Experimental setup for the induction of a blunt muscle trauma on left *extensor iliotibialis anticus*. (A) A weight with a mass of 40 g was dropped down by removing a pin over an effective drop height of 104 cm in order to induce a blunt skeletal muscle injury. (B) A spacer of 3 mm prevented the impact to break the bone by limiting the depth of penetration. The left leg of the mouse was placed onto the scaffold, whereas the wedge was placed on top of the *extensor iliotibialis anticus* muscle.
