# Supplementary material for: Diet-Induced Obesity Affects Muscle Regeneration After Murine Blunt Muscle Trauma—A Broad Spectrum Analysis
Source: Front Physiol. 2018 Jun 5;9:674. doi: 10.3389/fphys.2018.00674 (PMC5996306; doi:10.3389/fphys.2018.00674)
Supplement: Supplementary file 6 [file Image_2.pdf]

# Diet-induced obesity affects muscle regeneration after murine blunt muscle trauma – a broad spectrum analysis

Pengfei Xu[1†], Jens-Uwe Werner[1†], Sebastian Milerski[1], Carmen Hamp[1], Tatjana Kuzenko[1], Markus Jähnert[2], Pascal Gottmann[2], Luisa de Roy[3], Daniela Warnecke[3], Alireza Abaei[4], Annette Palmer[5], Markus Huber-Lang[5], Lutz Dürselen[3], Volker Rasche[4], Annette Schürmann[2], Martin Wabitsch[6]*, Uwe Knippschild[1]*

* Correspondence: Prof. Dr. Uwe Knippschild, uwe.knippschild@uniklinik-ulm.de and Prof. Dr. Martin Wabitsch, martin.wabitsch@uniklinik-ulm.de

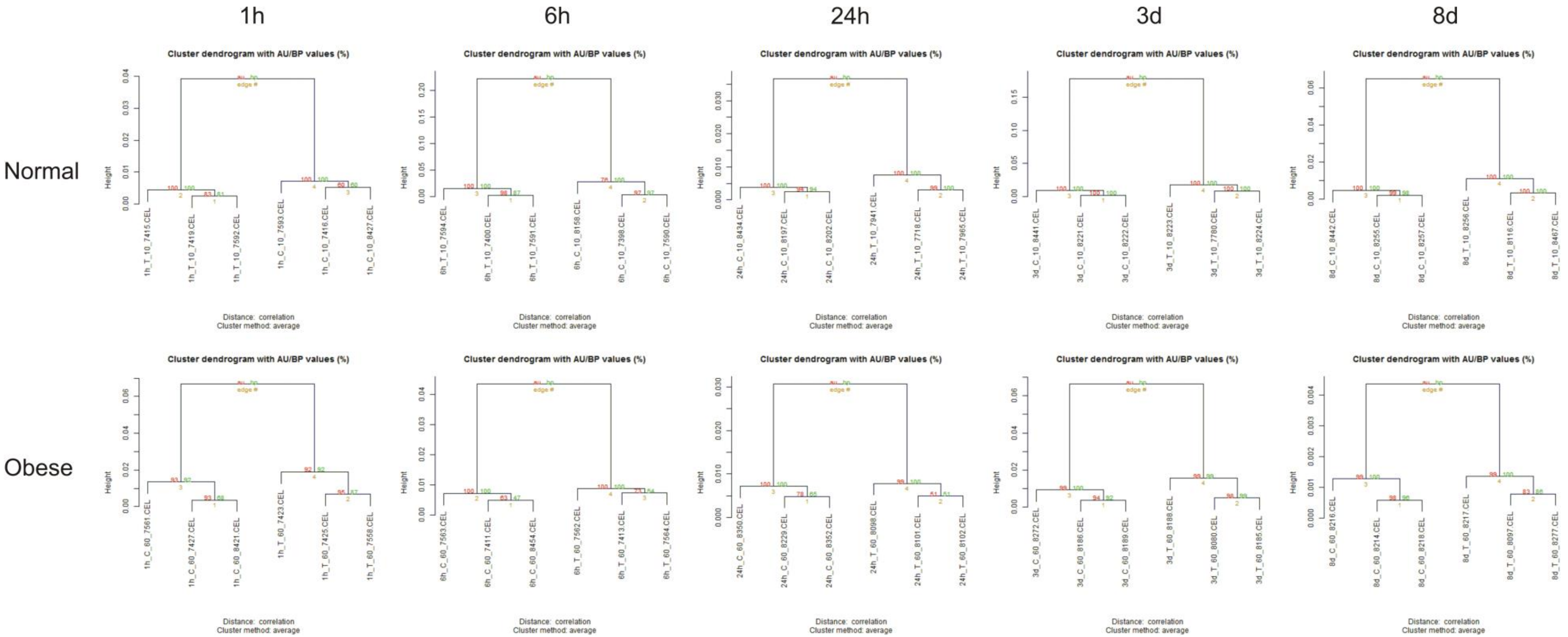


Sup. Fig. 2: Hierarchical cluster analysis (HCA) of muscle tissue from female control and trauma, lean and obese C57BL/6J mice between 1h to 8d post-injury. Red: AU = Approximately Unbiased; Green: BP = Bootstrap Probability, both in percent.
